# Transhemispheric cortex remodeling promotes forelimb recovery after spinal cord injury

**DOI:** 10.1172/jci.insight.158150

**Published:** 2022-06-22

**Authors:** Wei Wu, Tyler Nguyen, Josue D. Ordaz, Yiping Zhang, Nai-Kui Liu, Xinhua Hu, Yuxiang Liu, Xingjie Ping, Qi Han, Xiangbing Wu, Wenrui Qu, Sujuan Gao, Christopher B. Shields, Xiaoming Jin, Xiao-Ming Xu

**Affiliations:** 1 Spinal Cord and Brain Injury Research Group, Stark Neurosciences Research Institute, Department of Neurological Surgery, Indiana University School of Medicine, Indianapolis, Indiana, USA.; 2 Norton Neuroscience Institute, Norton Healthcare, Louisville, Kentucky, USA.; 3 Department of Biostatistics, Indiana University School of Medicine, Indianapolis, Indiana, USA.; 4 Mechanical Engineering, Worcester Polytechnic Institute, Worcester, Massachusetts, USA.

**Keywords:** Neuroscience, Therapeutics, Neurological disorders

## Abstract

Understanding the reorganization of neural circuits spared after spinal cord injury in the motor cortex and spinal cord would provide insights for developing therapeutics. Using optogenetic mapping, we demonstrated a transhemispheric recruitment of neural circuits in the contralateral cortical M1/M2 area to improve the impaired forelimb function after a cervical 5 right-sided hemisection in mice, a model mimicking the human Brown-Séquard syndrome. This cortical reorganization can be elicited by a selective cortical optogenetic neuromodulation paradigm. Areas of whisker, jaw, and neck, together with the rostral forelimb area, on the motor cortex ipsilateral to the lesion were engaged to control the ipsilesional forelimb in both stimulation and nonstimulation groups 8 weeks following injury. However, significant functional benefits were only seen in the stimulation group. Using anterograde tracing, we further revealed a robust sprouting of the intact corticospinal tract in the spinal cord of those animals receiving optogenetic stimulation. The intraspinal corticospinal axonal sprouting correlated with the forelimb functional recovery. Thus, specific neuromodulation of the cortical neural circuits induced massive neural reorganization both in the motor cortex and spinal cord, constructing an alternative motor pathway in restoring impaired forelimb function.

## Graphical abstract

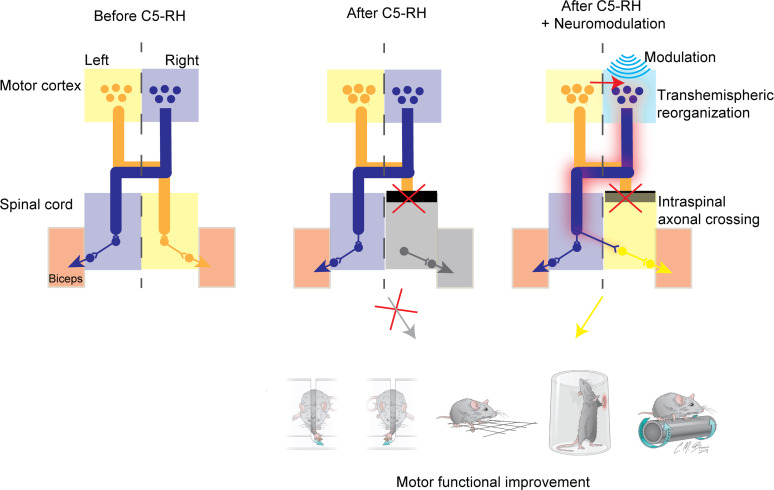

## Introduction

Neurological incomplete tetraplegia ranked first among spinal cord injuries (SCIs) clinically, according to the National Spinal Cord Injury Statistical Center’s Report (1), leading to severe motor impairments. Notably, injuries clinically classified as complete or American Spinal Injury Association Impairment Scale A (AIS-A) have been shown to have spared tissues (2). Due to the inhibitory environment following trauma and the lack of neuronal intrinsic capacity to regenerate, it is very difficult for axons to regenerate across a lesion gap and make functional connections to neurons caudal to the injury in the adult mammalian central nervous system (CNS) (3). Therefore, protection and modulation of the residual neural circuits are considered feasible strategies for improving functional recoveries after SCI (4, 5).

Reorganization of neural circuits in the motor cortex rostral to SCI remains understudied. Electrical intracortical microstimulation (ICMS) have traditionally been applied to map somatic motor and sensory representations clinically and experimentally (6). Several cortical motor representations could be organized according to classes of behavior (7). Recently, a novel rapid automated motor mapping based on optogenetic technology has been developed to map cortical neural circuits in a more consistent and quantitative way (6, 8). The cortical circuits for skilled forelimb function have been well deconstructed using both optogenetic mapping and tracing techniques in rodents without injury (9). After cervical SCI, cortical mapping showed a recruitment of the hindlimb area to control the forelimb (10). Similarly, incorporation of the corticospinal hindlimb area into the forelimb area after thoracic SCI was demonstrated using tracers and blood oxygen level–dependent functional MRI (BOLD-fMRI) (11). Thus, understanding and correlating the reorganization of both the cortical and spinal regions may provide new insights into neuromodulation-mediated functional recovery and facilitate the development of new targeted therapeutics.

Neuromodulation, a neuronal activity-based approach such as electrical stimulation, was shown to rebuild neural circuitry in the lumbar spinal cord after thoracic SCI (5, 12). The rationale behind this is the presence of a central pattern generator (CPG) located in the lower thoracic and upper lumbar region. Targeting this CPG using a stimulation strategy at multiple levels of the CNS shows promise in treating the CNS conditions. For example, motor cortex activation by deep brain stimulation (DBS) to the midbrain, or direct lumbar spinal cord activation by electrical stimulation, promoted the plasticity of activated pathways to form new neural circuitry, resulting in hindlimb locomotor functional recovery (12–16). At the cervical level, interestingly, electrical stimulation of the intact motor cortex also promoted forelimb skilled recovery in an experimental unilateral pyramidotomy model (17). Although the presence of a cervical CPG is debatable, the presence of a special neural circuitry that modulate forelimb function may exist.

Although most SCIs occur at the cervical level, fewer studies have been conducted at this level. Here we used optogenetic technology, both as a tool to investigate the reorganization of the motor map in the cortex and as a specific neuromodulation strategy to selectively activate the map in a clinical-relevant severe cervical SCI model. Our purpose was to understand the role of reorganization of the intact corticospinal tract (CST), the main pathway projecting from the motor cortex to the spinal cord, in regulating the forelimb functional recovery after a unilateral cervical spinal cord hemisection. In this case, we can assume that the neurons we tested in the optogenetic mapping experiment are the same groups of neurons that receive optogenetic modulation, which reduce the overall variation during the experiment.

## Results

### A consistent right side hemisection model mimics the human Brown-Séquard syndrome.

**Figure 1:**
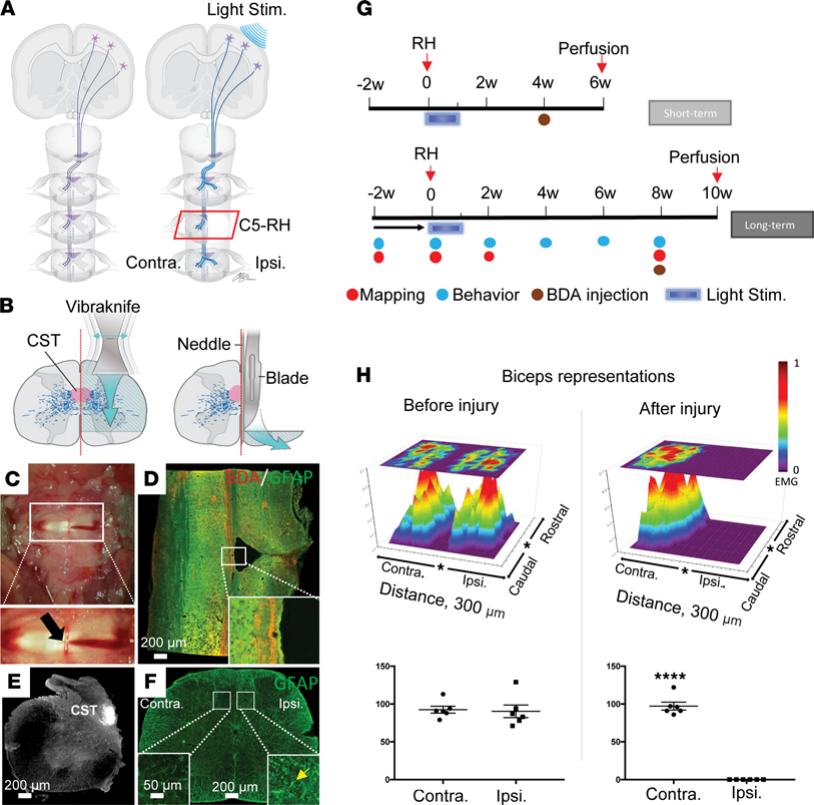
C5-RH completely eliminates the ipsilesional hemicord. (**A**) Schematic drawing shows the normal (left), and optogenetic-modulated (right) projection of the corticospinal tract. (**B**) Schematic illustration of a precise C5-RH. Left, a vibrating blade was used to cut the right spinal cord along the midline at C5; right, a modified needle, with a groove facing the lesion side. A fine blade was inserted into the groove and moved down to cut the spared ventral spinal tissue. (**C**) A C5-RH under a surgical microscope. An arrow indicates the midline and a Vibraknife cut on the right side. (**D**) A horizontal section shows a cut on the right side with a needle track next to the midline. BDA, CST (red); GFAP, astrocyte (green); Scale bar: 200μm. (**E**) A representative image shows a cross section of the remaining intact left hemicord; Scale bar: 200 μm. (**F**) GFAP immunofluorescent staining on a cross section of the spinal cord caudal to the C5 RH showed ipsilesional reactive astrogliosis (GFAP^+^, insert, yellow arrow). Such astrogliotic response was not found on the contralesional side; Scale bar: 200 μm; Insert: 50 μm (**G**) Timelines of the experiment.(**H**) Topographic representation of contralateral cortical recording of the CST on left/right side prior to and at 1 day after the C5-RH. Stars indicate bregma. EMG responses were represented by a hot spot. Statistical analysis of the dot numbers confirmed that ipsilesional control from the left motor cortex was complete disrupted, while the contralesional control from the right motor cortex was intact, indicating the preciseness and completeness of this injury model. Data were presented as mean ± SEM; *n* = 6 mice per group; 2-tailed paired Student’s *t* test; ****P < 0.0001.

Producing a consistent SCI model is essential for examining an injury mechanism or testing a therapeutic effect. In rodents, 95% of the CST axons are in the ventral aspect of the dorsal funiculus, namely the dorsal CST (18, 19). The left and right dorsal CSTs are located side by side within the dorsal funiculus, separated by a very thin posterior medium septum. To produce a consistent injury that completely transects the CST on one side and spares the one on the other side, a precise lateral hemisection through the midline is required. Thus, we first developed a well-controlled lateral hemisection model in the space between the Cervical 4 (C4) and Cervical 5 (C5) vertebrae, defined as a Cervical 5 right hemisection (C5-RH) (Figure 1A). This was done by stabilizing the cervical vertebrae prior to the injury (20) to ensure the cord is in an upright position, as we perform a right-sided hemisection stereotaxically with a VibraKnife attached to the Louisville Injury System Apparatus (LISA) device, which produces precise cutting accuracy (21) (Figure 1B). We choose laceration over other cutting methods due to the precision (0.01 mm) of the cutting and the minimization of the “extrusion” or “stretch” to the spinal cord. To preserve the vertebral bone structure, the cutting was made through the temporary-emerged gap between C4 and C5 vertebrae without laminectomy (Figure 1C). To reduce the potential heat damage to the spinal cord that might be generated by the vibration, we added saline to the surface of the spinal cord during the whole cutting process. The potentially spared spinal cord on the right side was further incised using a self-made thin blade and the left spinal cord was protected by a modified needle during the cutting (Figure 1B). The completeness of the C5-RH was confirmed in several ways. First, it was confirmed by direct observation under a surgical microscope (Figure 1C). Second, on a horizontal section of the spinal cord, the right hemicord was shown to be cut on the right side and dorsal CST axons on the contralesional side, labeled by biotinylated dextran amine (BDA), remained uncut, (Figure 1D) which can be appreciated in the insert. Third, a cross section through the injury epicenter showed that only the contralesional (left) hemicord was spared with a complete preservation of the intact CST, labeled with BDA (Figure 1E), whereas the ipsilesional (right) hemicord was completely removed by the injury. Fourth, in the spinal cord below the injury, increased astrogliotic responses (GFAP-immunoreactive) were found only on the ipsilesional side as a result of CST axotomy after the C5-RH (Figure 1F, yellow arrow) as compared with the lack of astrogliotic response on the contralesional side where CST axons were uninterrupted. Thus, both horizontal (Figure 1D) and transverse sections (Figure 1E) through the lesion epicenter show the interruption of the ipsilesional CST while leaving the contralesional CST uninterrupted. Functionally, optogenetic stimulation with a laser beam on one side of the motor cortex to elicit an electrical response from the opposite side of the anterior biceps, or vice versa, was performed to determine the continuity of the CST from one side of the motor cortex to the opposite side of the forearm muscle after the C5-RH (Supplemental Figure 1, A and B; supplemental material available online with this article; https://doi.org/10.1172/jci.insight.158150DS1). The number of stimulated spots at the primary motor cortex that resulted in muscle motor end-plate action potentials were mapped and plotted as spot numbers (Figure 1H). Spot numbers were the same between the 2 sides before the injury (Figure 1H; *P* = 0.85). However, the numbers were reduced from 90 prior to the injury to 0 after the injury on the ipsilesional side (Figure 1H; *P* < 0.0001) as compared with the unchanged contralesional side. Importantly, behavior assessments showed no change to the contralesional (left) forelimb as reflected in the pellet retrieval test (Supplemental Figure 2) and grid walking (see below). Thus, the hemisection model minimizes injury variation and offers a unique opportunity to assess cortical map changes, anatomical sprouting of the CST axons, and functional outcomes after the C5-RH.

### Optogenetic neuromodulation induces a significant cortical transhemispheric reorganization.

**Figure 2:**
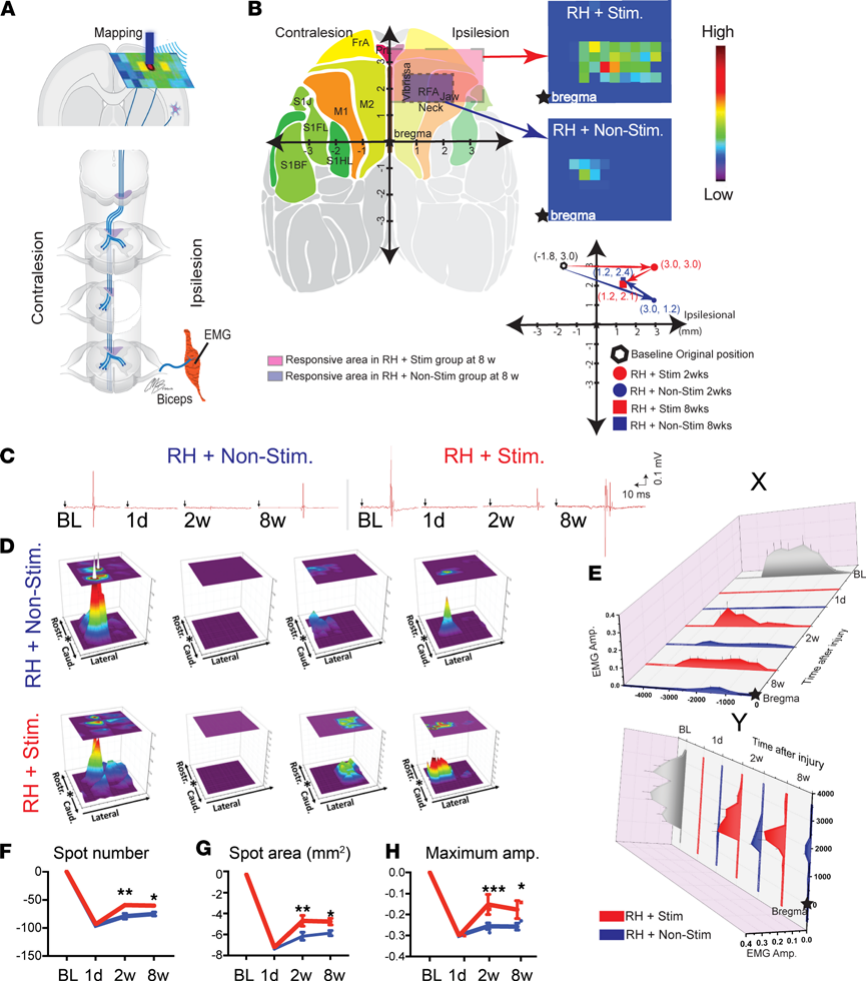
Optogenetic stimulation modulates transhemispheric remapping which enhances electrophysiological responses. (**A**) Schematic drawing illustrates how the mapping was conducted; (**B**) Mice with optogenetic stimulation showed enhanced transhemispheric cortical map shift. Left, cortical map reorganization at 8 weeks after a C5-RH in mice with or without stimulation. Pink square, the responsive mapping area at 8 weeks after the C5-RH with optogenetic modulation. Blue square, the responsive mapping area at 8 weeks after C5*RH without optogenetic modulation. RFA, rostral forelimb area. Right, representative heat map shows the increased response in optogenetic stimulation group at 8 weeks after the C5-RH compared with the nonstimulation group. Right bottom, the coordinates of the spot with maximum amplitude at different time points. (**C**) Representative EMG traces with an arrow indicating the onset of laser stimulation in C5-RH mice. (**D**) Representative heat maps of the ipsilesional biceps recording after laser stimulation at the ipsilesional motor cortex in both the stimulation and nonstimulation groups at different time points after injury. (**E**) Maximum amplitude projection on x and y axes. (**F–H**) Quantitative comparison of spot number, response area, and amplitude between the stimulated and nonstimulated groups. *n* = 6 per group. Data are presented as the mean ± SEM; statistical evaluation was carried out with 2-way ANOVA repeated measure followed by Tukey’s multiple comparisons test; *P < 0.05, **P < 0.01, ***P < 0.001.

Using the C5-RH model and optogenetic mapping, we first determined whether the ipsilesional (right) motor cortex, originally controlling the contralesional (left) forearms, was enrolled in controlling the ipsilesional forearms. An array of 180 points were rapidly stimulated on the ipsilesional cortex, and the electromyogram (EMG) from both ipsilesional and contralesional anterior biceps were recorded during the optogenetic stimulation (Figure 2A, Supplemental Figure 1A, and Supplemental Figure 3A). The maximum amplitude on x and y axes was analyzed in both nonstimulation and stimulation animals that received C5-RH at different time points. The connectivity of the intact motor cortex to the nonlesioned (left) forelimb and lesioned (right) forelimb were examined separately, as characterized by ipsilesional and contralesional recordings (Supplemental Figure 3, B–D, Supplemental Figure 4). In the ipsilesional recording (Figure 2B), the dots with maximum amplitude from the stimulation and nonstimulation groups were plotted on the mouse brain map (modified from ref. 22). A total of 3 parameters were derived from the raw data: spot number (Figure 2F and Supplemental Figure 3E), spot area (Figure 2G and Supplemental Figure 3F) and amplitude (Figure 2H and Supplemental Figure 3G). Spot number is defined as the number of stimulated spots on the cortex that gave an EMG response above threshold. Spot area is defined as the total dimension of the area that gave an EMG response to laser stimulation. Amplitude is defined as the amplitude observed along a coordinate plane in the x and y axes. In the case of recording ipsilesional forelimb, the forelimb EMG could not be elicited by stimulation at 1 day after the C5-RH in either the x or y axis (Figure 2, C–E).

Before injury, we observed functional connectivity of the ipsilesional (right) cortex and ipsilesional (right) forelimb (Figure 2, D and E), which is contrary to the understanding that the right side of the cortex controls the left side of the body. We hypothesized that the signal from the right cortex may travel via the corpus callosum to activate the left cortex whose axons form the right CST (after the pyramidal decussation) to control the right forearm activity. To test this possibility, a corpus callosotomy (CC) was performed (Supplemental Figure 4, A and B). Following this procedure, no functional connectivity was recorded between the ipsilesional (right) motor cortex and ipsilesional (right) forelimb (Supplemental Figure 4A), supporting our hypothesis. Furthermore, the spot number immediately dropped to 0 after the C5-RH and corpus callosotomy in the ipsilesional forearm recording (Supplemental Figure 4A), indicating the existence of cortico-cortical connections via the corpus callosum in the normal brain. Thus, the functional improvement of the impaired forelimb might be dependent on the newly formed neural circuits that may involve the ipsilesional cortex, the contralesional cortex, and the ipsilesional spinal cord.

We observed a spontaneous transhemispheric remapping of cortical motor output at 2 and 8 weeks after injury (Figure 2). Remarkably, the remapping occurred in animals receiving optogenetic stimulation (Figure 2, B–D). Despite limited spontaneous recovery in the nonstimulation group, the spot number, spot area, and amplitude were significantly increased in the stimulation group at 2 and 8 weeks after the C5-RH (Figure 2, F–H). In the contralesional recording, reflecting the normal function of the contralesional forelimb, no difference in the spot number and spot area was found (Supplemental Figure 3, E and F). Collectively, these results show that the contralesional CST forms functional connectivity with the ipsilesional forelimb as early as 2 weeks after injury, and this connectivity was sustained for at least 8 weeks following the injury. The amplitude was analyzed on x and y axes to observe the strength of the connectivity formed. In the ipsilesional recording, we observed a stronger signal recording in the stimulation group compared with the nonstimulation group (Figure 2, C and H), indicating that the stimulation promotes stronger functional connectivity of the motor cortex with the ipsilesional spinal cord. Although there was no significant difference in the contralesional spot number and spot area (Supplemental Figure 3, E and F), the amplitude was significantly increased in the stimulation group (Supplemental Figure 3G), suggesting that cortical stimulation of the intact motor cortex also enhances the activity of existing neural circuits in the contralesional forearm. In addition, the latency analysis showed no significant changes in either ipsilesional or contralesional recordings among groups (Supplemental Figure 5). Thus, the optogenetic cortical mapping reveals regaining of the cortical control over the paralyzed right forelimb after the C5-RH with or without neuromodulation, offering direct evidence for marked remodeling of functional neurocircuitries after selective cortical neuromodulation.

### Optogenetic stimulation promotes contralesional intact CST axons to sprout across the midline to innervate the ipsilesional hemicord at 6 weeks after injury, a short-term observation.

**Figure 3:**
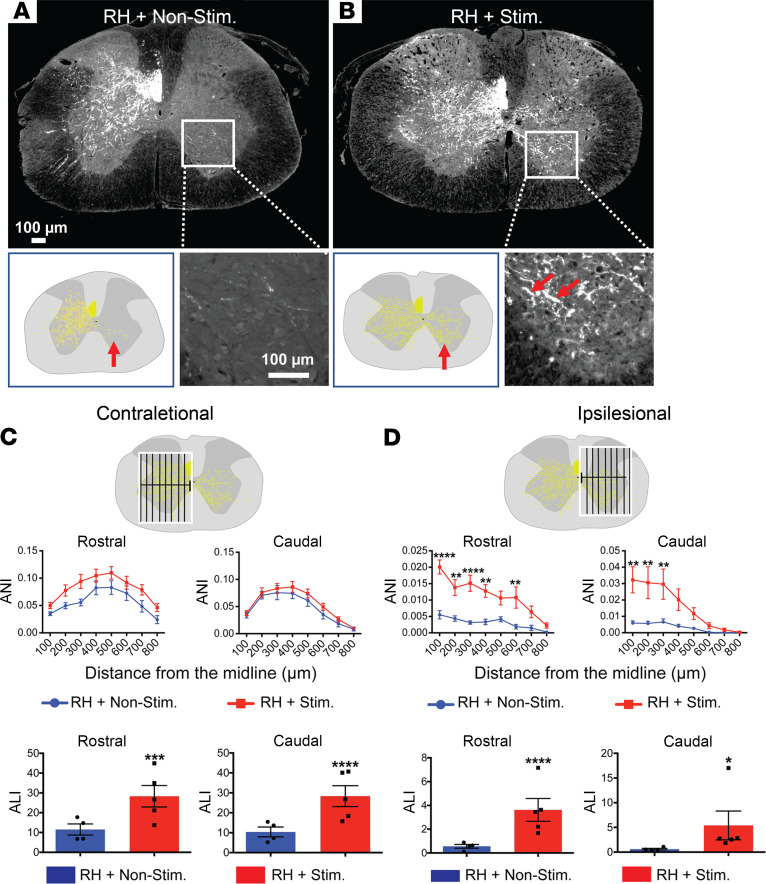
Transcranial optogenetic stimulation promotes intact contralesional CST axons to sprout into the ipsilesional hemicord at 6 weeks after the C5-RH. (**A** and **B**) Representative cross sections caudal to the C5-RH shows that the contralesional intact CST, labeled with BDA, located within the ventral portion of the dorsal funiculus. Robust CST axonal sprouting across the midline was observed only after optogenetic stimulation in **B** as compared with the nonstimulation case in **A**. High magnification of boxed areas further demonstrated the presence in B or absence in A of crossed CST terminals in the ipsilesional hemicord. Representative Neurolucida drawings show the uncrossed and crossed CST axons (yellow) in a nonstimulated case in A or a stimulated case in B. (**C**) Quantification of the intact CST axons projecting to the contralesional side (normal projection) at levels both rostral and caudal to the injury. Optogenetic stimulation enhanced axonal length of the CST on the contralesional side. (**D**) Quantification of intact CST axons projecting to the ipsilesional side (denervated side) at levels both rostral and caudal to the injury. Robust CST axonal sprouting at various distances from the midline was only observed in cases receiving optogenetic stimulation as compared with the nonstimulation cases at levels rostral and caudal to the injury. Data were presented as the mean ± SEM; *n* = 5 per group; 2-way ANOVA followed by Tukey’s multiple comparisons test; *P < 0.05, **P < 0.01, ***P < 0.001, ****P < 0.0001. Scale bars: 100 μm.

Axons of the layer V motor neurons in the motor cortex descend, via pyramidal decussation, to the contralateral side of the spinal cord in mammals. We next examined the sprouting of the intact, contralesional CST in the spinal cord at 6 weeks after the C5-RH. The CST axons were anterogradely labeled with BDA. After perfusion and tissue harvesting, mouse spinal cord tissues were cut transversely above and below the injury for visualization of the labeled CST axons that crossed the midline to innervate the ipsilesional hemicord (Figure 3, A and B). All BDA-labeled CST axons were manually drawn using a Neurolucida System (MBF Bioscience) in a double-blinded manner (Figure 3, A and B), and the results were normalized by the BDA-labeled CST axon number counted at the C2 level. Our data show that optogenetic stimulation promoted robust sprouting of the intact, contralesional CST axons to the ipsilesional gray matter at levels both rostral (Supplemental Figure 6, A and B), and caudal to the injury (Figure 3, A and B, and Supplemental Figure 6B) as compared with the nonstimulation group. Although there was spontaneous sprouting in the nonstimulation group, the stimulation group showed much stronger sprouting across the midline. These axons extended deeply into the intermediate and ventral gray matter (Figure 3B). Sprouting was quantified using 2 measurements: the axon length index (ALI) as an indicator of general axonal elongation and the axon number index (ANI) as an indicator of axonal branching, spaced at every 100 μm from the midline (Figure 3, C and D). On the contralesional side, optogenetic stimulation led to a significant increase in ALI, but no difference in branching (ANI) was observed (Figure 3C). On the ipsilesional side, however, optogenetic stimulation significantly enhanced both axon length and branching at levels rostral and caudal to the injury (Figure 3D). These data collectively suggest that optogenetic stimulation of the intact CST on the contralesional side promotes robust axonal sprouting across the midline to innervate the denervated ipsilesional spinal gray matter.

### Optogenetic stimulation maintains intraspinal CST sprouting in the spinal cord for at least 10 weeks after injury, a long-term observation.

**Figure 4:**
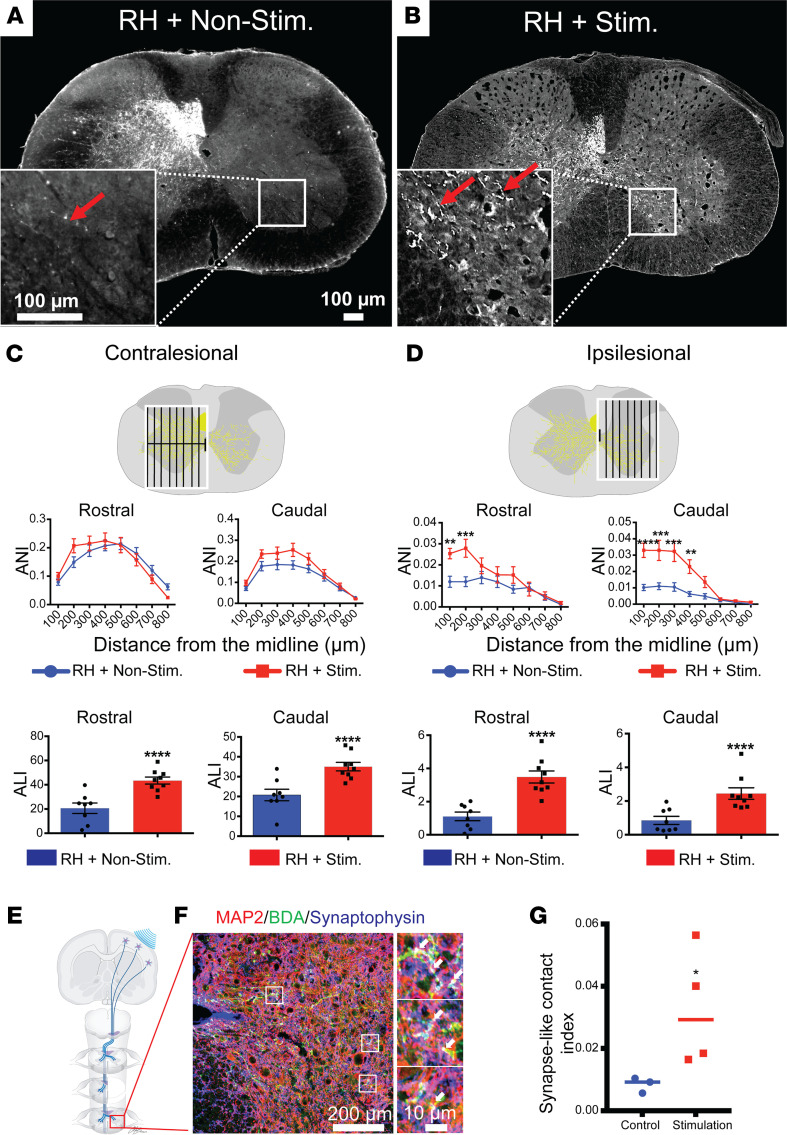
Transcranial optogenetic stimulation promotes intact contralesional CST axons to sprout into the ipsilesional hemicord at 10 weeks after the C5-RH, a long-term observation. (**A** and **B**) Representative spinal cord cross sections caudal to a C5-RH show that the contralesional CST axons sprouted across the midline to innervate the ipsilesional hemicord in the RH + Stim group (*n* = 9) as compared with the RH + Nonstim group (*n* = 8). Scale bars: 100 μm (**C**) Quantification of the intact CST axons projecting to the contralesional (left) side at levels both rostral and caudal to the injury. Like the short-term (6 weeks) group, optogenetic stimulation enhanced axonal length of the CST on the contralesional side. (**D**) Quantification of intact CST axons projecting to the ipsilesional (right) side at levels both rostral and caudal to the injury. Robust CST axonal sprouting across the midline at various distances was only observed in cases receiving optogenetic stimulation as compared with the nonstimulation cases at levels rostral and caudal to the injury. (**E**) schematic drawing shows that ipsilesional spinal cord caudal to the lesion was examined. (**F**) BDA-labeled CST axons crossed the midline to the ipsilesional spinal gray matter and contacted the target neurons. At higher magnification, triple labeling of BDA-labeled CST axons (green), MAP2-labeled neurons (red) and dendrites, and a presynaptic marker synaptophysin (blue) were found (white, arrows), indicating new synaptic formation between sprouted CST axons and target neurons in the ipsilesional gray matter. Scale bars: 100 μm (left), 10 μm (right) (**G**) Statistical comparison of synapse formation between the nonstimulated group and stimulated groups was shown. Data are presented as the mean ± SEM; statistical evaluation was carried out with 2-way ANOVA repeated measure followed by Tukey’s multiple comparisons test; *P < 0.05, **P < 0.01, ***P < 0.001, ****P < 0.0001. Stim, stimulation; Nonstim, nonstimulation.

We then asked whether the optogenetic stimulation-mediated anatomical reorganization would maintain long term in the ipsilesional gray matter. Therefore, we allowed mice to survive for 10 weeks after the C5-RH (Figure 4). BDA was injected at 8 weeks after injury, and spinal cord tissues were harvested at 10 weeks after injury (Figure 1G). In the contralesional spinal cord, the axon length was significantly increased (Figure 4C, ALI) in the optogenetic stimulation group whereas axonal branching (Figure 4C, ANI) showed no difference at levels both rostral and caudal to the injury, similar to what was observed at 6 weeks following the injury. In the ipsilesional spinal cord, increased ALI and branching (ANI) of the CST were observed in the stimulation group at levels both rostral and caudal to the injury (Figure 4D and Supplemental Figure 7, A and B).

To further dissect the influence of the optogenetic stimulation on axonal growth in naive animals, we performed the same stimulation and axon analysis in sham mice (Supplemental Figure 8). A similar substantial rise in CST ALI without significant change of axonal branching (ANI) was observed on the contralateral hemicord. On the ipsilateral hemicord (Supplemental Figure 8, A and B), although ALI was significantly increased in the stimulation group, no axonal branching (ANI) was observed as compared with the nonstimulation group. These results indicate that the C5-RH contributes to axonal branching. Overall, these results indicate that the effect of optogenetic stimulation can be sustained for a long time after SCI. Although the CST axonal elongation is significantly enhanced by optogenetic stimulation in both the ipsilesional and contralesional hemicords, optogenetic modulation on axonal branching occurs only in the hemicord that receives the C5-RH.

### Optogenetic stimulation enhances synaptogenesis in the contralesional spinal cord.

Since SCI results in significant reduction in the number of synapses caudal to the lesion (23) and since new synaptic formation is essential for improving functional recovery, we next examined whether sprouted CST axons formed synaptic contacts with neurons in the ipsilesional gray matter after optogenetic stimulation. Spinal cord tissues were harvested at 10 weeks after injury and were examined at levels caudal to the lesion site. Colocalization of BDA-labeled CST axons with synaptophysin (present in presynaptic vesicles) and microtubule-associate protein 2 (MAP2), a neuronal marker, were found in the ipsilesional intermediate and ventral gray matter after optogenetic stimulation, suggesting the formation of new synapses between the sprouted CST axons and ipsilesional dendrites (Figure 4, E and F). As expected, the stimulation group had a significantly higher number of synaptic contacts that triple-labeled with BDA, MAP2, and synaptophysin as compared with the nonstimulation group (*P* = 0.0416) (Figure 4G). These results indicate that optogenetic-stimulated CST axons can form synaptic contacts in the ipsilesional spinal gray matter in areas where interneurons and motoneurons are located after the C5-RH, providing evidence for the formation of an alternative corticospinal pathway that mediates ipsilesional forelimb recovery.

### Optogenetic stimulation of the ipsilesional motor cortex promotes ipsilesional forelimb skilled function and sensorimotor coordination recovery following the C5-RH.

**Figure 5:**
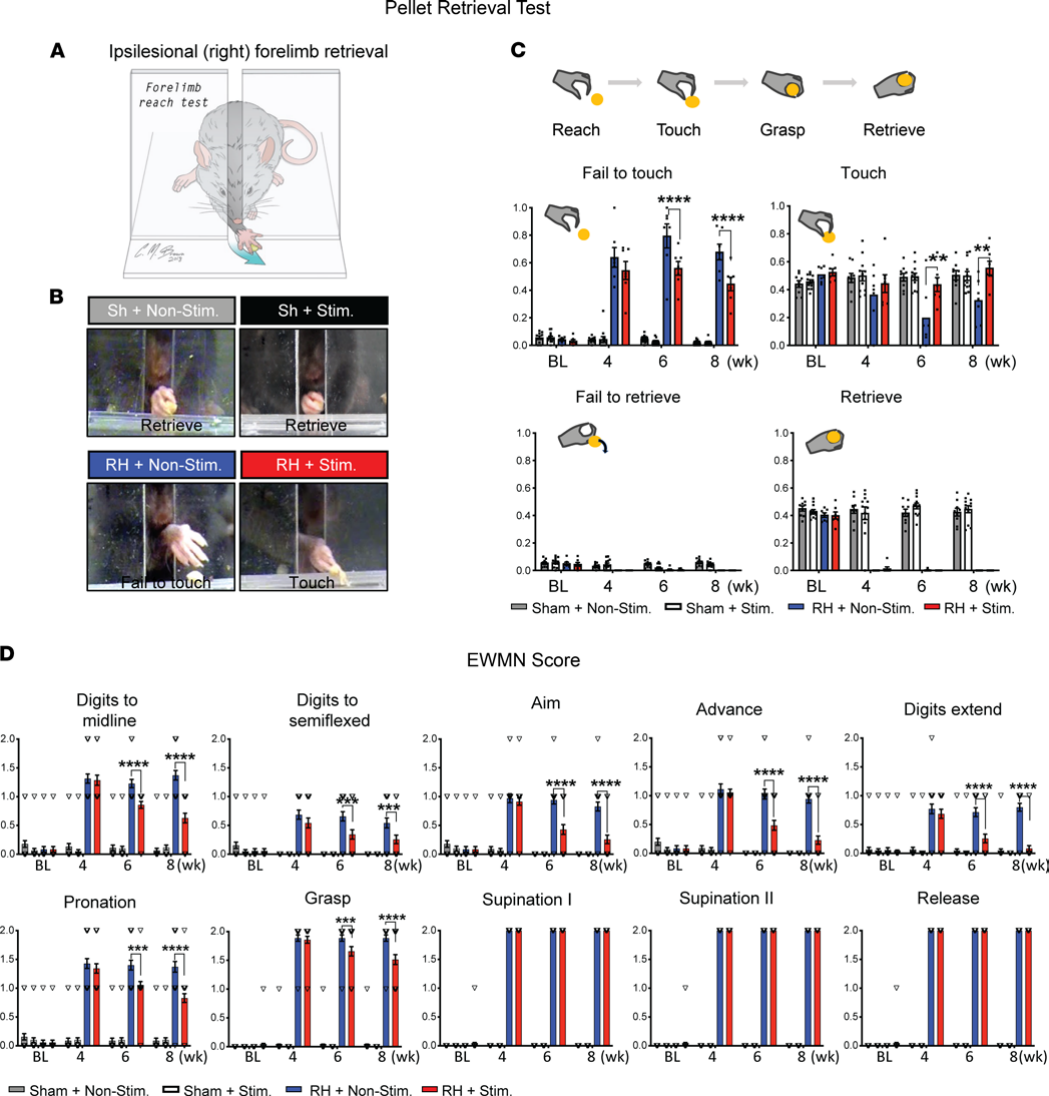
Optogenetic stimulation improves ipsilesional task-specific single pellet retrieval function after the C5-RH. (**A**) Schematic drawing shows the method of the ipsilesional (right) forelimb dexterous function tested in a single pellet retrieval task. Millets seeds were put on the right side in front of the chamber slit, preventing the contralesional (left) forelimb from reaching them. (**B**) Representative images show examples of pellet retrieval in 4 experimental groups. (**C**) A total of 4 consecutive steps towards a complete pellet retrieval process depending on difficulty levels. In the 4 parameters examined, optogenetic stimulation significantly reduced the Fail to touch and improved Touch as compared with the nonstimulation group at 6 and 8 weeks after the C5-RH. Stim, stimulation; Nonstim, nonstimulation. (**D**) Scores for each of the 10 movement components of the single pellet retrieval task. The reduced score is shown in stimulation group in digits to midline, digits semiflexed, aim, advance, digits extend, pronation, and grasp, reflecting an improved skilled functional recovery to an extent. *n* = 5–7 per group. Data were presented as the mean ± SEM; 2-way ANOVA followed by Tukey’s multiple comparisons test; **P < 0.01, ***P < 0.001, ****P < 0.0001. **)**

We next asked whether the optogenetic stimulation of the ipsilesional motor cortex (via the intact, contralesional CST) promote ipsilesional forelimb functional recovery. To determine whether augmenting the activity in the contralateral motor cortex improves ipsilesional forelimb dexterous function, task-specific single pellet retrieval test was performed in both the ipsilesional and contralesional forelimbs (Figure 5A and Supplemental Figure 2A). In this experiment, mice received the C5-RH followed by 1 week of intense optogenetic stimulation. The pellet retrieval task was performed at 4, 6, and 8 weeks after injury (Figure 1G) (24). A pellet retrieval task can be divided into reach, touch, grasp, and retrieve steps (Figure 5C). A total of 4 groups of mice with various treatments were subjected to the test (Figure 5B). To assess mice dexterity after injury, 4 parameters were used to score the reaching attempts according to our previous protocol (25). They include fail to touch, touch, fail to retrieve, and retrieve (Figure 5C). In this test, both ipsilesional forelimb (Figure 5A) and contralesional forelimb (Supplemental Figure 2A) were tested with pellet retrieval assessment, respectively. We found that optogenetic stimulation significantly improved ipsilesional forelimb skilled function after the C5-RH as compared with the nonstimulation group in the categories of fail to touch and touch (Figure 5C). A significant decline of fail to touch (RH + Nonstimulation versus RH + Stimulation: *P* < 0.0001 at 6 weeks; *P* < 0.0001 at 8 weeks) and increase of touch (RH + Nonstimulation versus RH + Stimulation: *P* = 0.0012 at 6 weeks; *P* = 0.0018 at 8 weeks; Supplemental Video 1) were achieved with optogenetic stimulation. However, neither group was able to perform the retrieve task (Figure 5C), indicating that the optogenetic stimulation alone was not sufficient to induce full-range forelimb recovery. In the Sham + Nonstimulation and Sham + Stimulation groups, there were no statistically significant differences in pellet retrieval test of both forelimbs (Figure 5C and Supplemental Figure 2, A and B). Thus, stimulation alone without injury does not improve inherent forelimb motor function.

To further reveal the movements associated with the pellet-retrieval task, we performed Eshkol-Wachmann Movement Notation (EWMN) scoring (26), which documents the pellet-retrieval task into consequential 10 steps (Figure 5D). Optogenetic-stimulated animals showed significant improvement in “digits to midline,” “digits semiflexed,” “aim,” “advance,” “digits extend,” “pronation,” and “grasp” as compared with nonstimulated animals. No significance was found in “supination I,” “supination II,” and “release” between the 2 groups, due to the failure to retrieve. These results further reveal that optogenetic stimulation has promoted recovery of task-specific pellet-retrieval movement to a certain extent. The full dexterous recovery, however, was not achieved, consistent with the original 4 parameter measures (Figure 5C).

**Figure 6:**
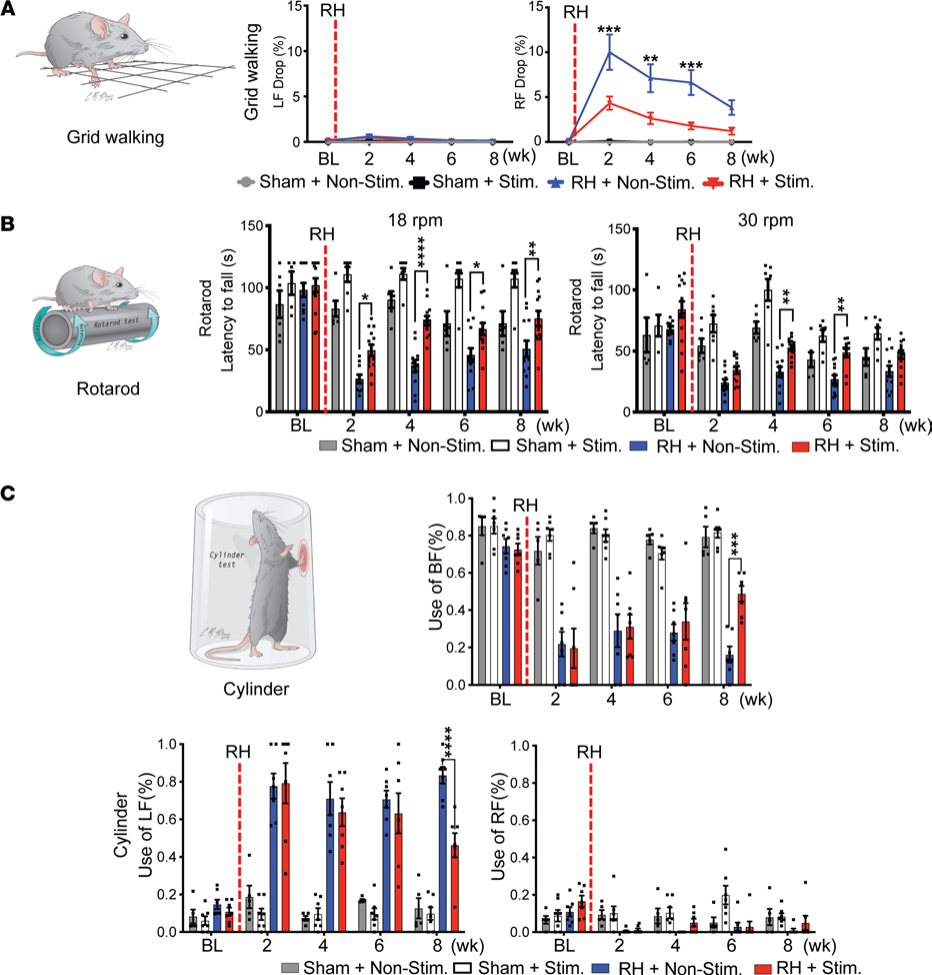
Optogenetic stimulation improves other forelimb less-specific skilled and locomotor functional recovery. (**A**) Grid walking test showed no foot drops (as mistakes) on both the left (LF) and right (RF) forelimbs in the sham groups. Significantly increased foot drops were found in the ipsilesional forelimbs of the RH + Nonstim group at 2, 4, and 6 weeks after injury as compared with the RH + Stim group. *n* = 5–14 per group. (**B**) Rotarod test was performed at 2 different speeds, i.e., 18 and 30 rotations per minute (rpm). Optogenetic stimulation showed improvements in both the slow (18 rpm) and fast (30 rpm) speeds as compared with the nonstimulation control after the C5 RH. *n* = 6–14 per group. (**C**) Cylinder test showed that optogenetic stimulation significantly reduced the usage of the left hand (intact hand) and increased the usage of both hands (both the intact and impaired hands) at 8 weeks after injury, indicating improvement of the impaired hand function on the right side. *n* = 5–7 per group. Data were presented as the mean ± SEM; 2-way ANOVA followed by Tukey’s multiple comparisons test; *P < 0.05, **P < 0.01, ***P < 0.001, ****P < 0.0001. RF, right forelimb; LF, left forelimb; BF, both forelimbs; Stim, stimulation; Nonstim, nonstimulation.

The grid walking test was used to evaluate the sensorimotor coordination of the forelimbs and hindlimbs. This test requires animals to accurately place their limbs on the bars while crossing grids. Although this test is “less precise” than the pellet retrieval test, it indicates paw placement accuracy, which is another skilled functional assessment for forelimb recovery (Figure 6A) (27). In this test, mice with optogenetic stimulation showed a significant decrease in ipsilesional (right) forelimb drops at 2, 4, and 6 weeks after the C5-RH as compared with the nonstimulation group (Figure 6A), indicating that optogenetic stimulation can enhance skilled forelimb recovery on the ipsilesional side (RH + Nonstimulation versus RH + Stimulation: *P* < 0.0001 at 2 weeks; *P* = 0.0013 at 4 weeks; and *P* = 0.0004 at 6 weeks). However, there was no statistically significant difference at 8 weeks following injury between the 2 groups (*P* = 0.1244), likely due to a gradual spontaneous recovery occurring in the nonstimulation group at the eighth week.

The rotarod test was used to evaluate motor coordination and forelimb grip on a rotating cylindrical bar (Figure 6B) (28). The mice were trained to learn to walk on the rotating bar before experiments. The optogenetic stimulation group was able to stay significantly longer than the nonstimulation group on the rotating rod at 2, 4, and 8 weeks after injury at the speed of 18 rpm (Figure 6B; RH + Nonstimulation versus RH + Stimulation: *P* = 0.0381 at 2 weeks; *P* < 0.0001 at 4 weeks; and *P* = 0.0194 at 8 weeks). When the rotation speed was increased to 30 rpm, the stimulation group still stayed longer on the rotation rod than the nonstimulation group at 4 and 6 weeks after injury (Figure 6B; RH + Nonstimualtion versus RH + Stimulation: *P* = 0.0081 at 4 weeks; *P* = 0.0063 at 6 weeks). No significant changes were found in the sham-operated group in the presence or absence of optogenetic stimulation at all time points that were studied.

We next evaluated whether cortical optogenetic stimulation could improve other locomotor functions. We selected 2 additional functional assessments, i.e., cylinder and overground locomotor tests, to study sensorimotor and motor function following the C5-RH (Figure 6C and Supplemental Figure 9). For the cylinder test, forelimb activity was recorded as the mouse reared and contacted the wall of the cylinder with either right (ipsilesional), left (contralesional), or both paws. We defined the forelimb asymmetry as the mouse favoring the left side, i.e., the nonlesioned side, after the C5-RH. The optogenetic stimulation group increased ipsilesional forelimb usage at 8 weeks after SCI (Figure 6C).

General locomotion was further tested using a smart-cage system, which evaluated movement velocity and distance traveled within a compartment (29). Cortical optogenetic stimulation significantly increased moving distance (Supplemental Figure 9, A and B), whereas the optogenetic stimulation did not promote the mice to move faster (Supplemental Figure 9C), indicating that the mice traveled longer distances after optogenetic stimulation as compared with the nonstimulation group. Thus, our findings provide evidence showing that enhanced remodeling of spared neural circuits in the motor cortex and the CST improves motor recoveries following the C5-RH.

### Close correlation between neuromodulation, intraspinal CST sprouting, and functional recovery.

**Figure 7:**
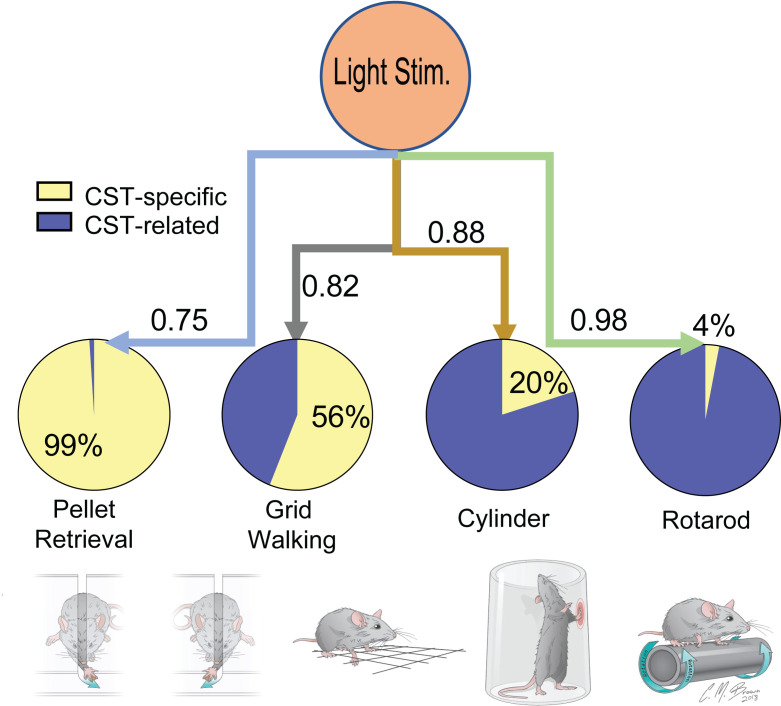
Correlation analysis shows various degrees of correlation between optogenetic stimulation and different behavioral outcomes. Optogenetic stimulation is positively related to different forelimb functional recoveries in various behavior tests. The correlation of the numbers shown next to the lines connecting light stimulation to the 4 behavioral outcomes are the correlation coefficients between optogenetic stimulation and behavioral outcomes. CST-specific effects (yellow in pie chart) are the percentages of change in R^2^ associated with optogenetic stimulation in behavior recovery outcomes when axonal sprouting below the injury site was included as an explanatory variable, representing the proportion in the effects of optogenetic stimulation on behavioral outcomes that were accounted for by axonal sprouting. CST-related effects (blue in pie chart) indicate the percentage of function recovery is mediated possibly through other descending pathways receiving CST projections in other regions of brain or spinal cord.

Next, we determined the correlations between optogenetic stimulation or ipsilesional CST sprouting and behavior outcomes. The correlation analysis is conducted blindly in a biostatistics lab at the Indiana University School of Medicine. In the pie chart, CST-specific motor improvements induced by optogenetic stimulation were positively correlated most strongly with results of the rotarod test (r = 0.98), followed by the cylinder (r = 0.88), grid walking (r = 0.82), and pellet retrieval (r = 0.75) tests (Figure 7). Interestingly, axonal sprouting after optogenetic stimulation was strongly correlated with pellet retrieval (99%, measured as the percentage of change in R squared when axonal sprouting below the injury site was added to the model as another intendant variable in addition to optogenetic stimulation), less strongly with grid walking (56%), but weakly with cylinder (20%). Although the improvement of rotarod was strongly correlated with optogenetic stimulation, it was not correlated with intraspinal CST axonal sprouting (4%, Figure 7). This indicates that an alternative pathway from the ipsilesional motor cortex, via the contralesional CST, to the ipsilesional forelimb motor system may be crucial for modulating the pellet retrieval function and the grid walking function, whereas the cylinder and rotarod functions are mediated by some unknown pathways or mechanisms. Such a functional alternative pathway from the ipsilesional motor cortex to the ipsilesional forearm is supported by anatomical, electrophysiological, and behavioral outcomes provided by the current study.

### Neuromodulation by optogenetic stimulation significantly increases neurotrophin brain-derived neurotrophic factor expression in both the cerebral cortex and spinal cord after the C5-RH.

**Figure 8:**
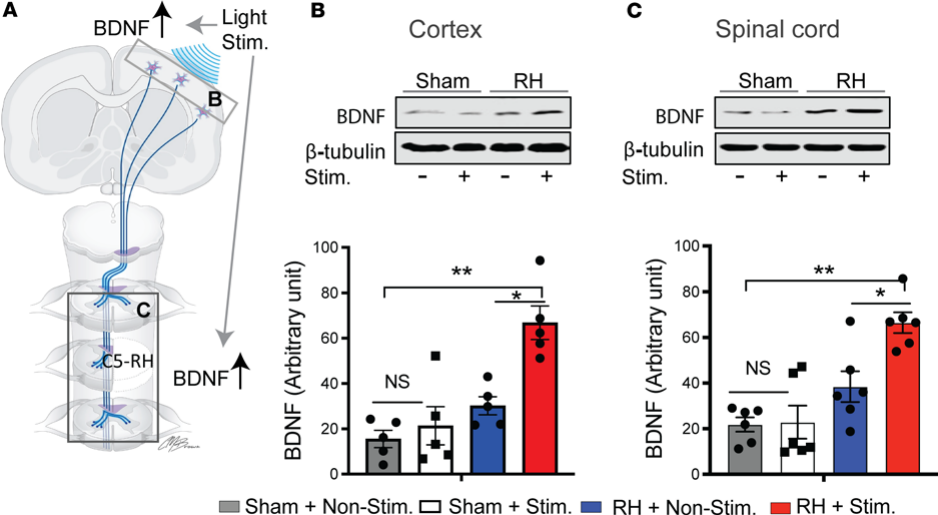
Optogenetic stimulation induces changes of BDNF in both the motor cortex and the injured spinal cord distal to the stimulation. (**A**) Scheme of the location of CNS tissue extracted for protein analysis. (**B** and **C**) optogenetic stimulation significantly increased BDNF expression in both the motor cortex and spinal cord after the C5 RH. No significant differences were found in sham animals between light-stimulated and nonstimulated groups. *n* = 5–6/group. Data are presented as the mean ± SEM; 2-tailed paired Student’s *t* test; *P < 0.05, **P < 0.01. Stim, stimulation; Nonstim, nonstimulation.

Last, we examined whether a neurotrophin brain-derived neurotrophic factor (BDNF) was expressed in the brain or spinal cord after optogenetic stimulation. BDNF has been shown to be closely linked to neuroplasticity (30, 31), such as axonal branching (32) and neural connection between the CST and spared descending interneurons (33). We compared ipsilesional motor cortex and spinal cord tissue at the lesion site (Figure 8A) between the stimulation and nonstimulation groups using Western blot analysis. Following the C5-RH, both the cortical and spinal cord BDNF expressions were significantly increased in the stimulation group as compared with the nonstimulation group (Figure 8, B and C). An optogenetic stimulation-induced increased level of BDNF expression was not found in the sham group with or without optogenetic stimulation. These results indicate that cortical optogenetic stimulation not only enhances BDNF expression at the site of cortical stimulation but also at the injured spinal cord of its projection, a long distance away from the light stimulation.

Thus, the C5-RH induces spontaneous neural circuits reorganization in the contralesional motor cortex and in the spinal cord. This reorganization can be further enhanced by selective optogenetic stimulation. Optogenetic-mediated neuromodulation occurs not only in the motor cortex, but also in the spinal cord, evidenced by increased axon length and branches in the denervated ipsilesional hemicord, leading to functional improvements including skilled forelimb and locomotive recoveries.

## Discussion

The purpose of this study was to understand how cortical transhemispheric neural reorganization affects the CST plasticity and recovery of forelimb function after a C5-RH. We demonstrated that several motor areas in the ipsilesional motor cortex (supposed to control the contralesional forelimb) are recruited to control the ipsilesional forelimb after a C5-RH. We also showed that optogenetic stimulation significantly improves the size and amplitude of the reorganized cortical map, leading to skilled forelimb and locomotor recovery. The motor map spread to adjacent regions after SCI has been previously observed (10, 11); however, the long-distance transhemispheric remapping and its functional importance had not been shown. We further found that neuromodulation by optogenetic stimulation not only induced beneficial reorganization in the motor cortex, but also facilitated intact CST axonal sprouting and synaptogenesis in the denervated ipsilesional hemicord. Thus, optogenetic neuromodulation of the motor cortex is sufficient to induce massive transhemispheric and trans-spinal neural reorganization, leading to meaningful functional improvement after a severe unilateral cervical SCI in adult mice.

One of our main purposes was to explore the role of cortex-oriented CST systems in modulating functional recovery following SCI. We showed that neuromodulation by optogenetic stimulation is precise and efficient in mediating corticospinal remodeling after SCI. Anatomically, the cerebral cortex consists of 6 layers that contain heterogeneous populations of neurons (and glial cells) with a variety of neurotransmitters, either excitatory or inhibitory, coordinately released. Whereas electrical stimulation activates all cell types at the same time, the optogenetic stimulation activates cortical pyramidal neurons, without interrupting other cell types, such as microglia, astrocyte, and oligodendrocyte, in the region. This makes the technique more precise, specific, and efficient as compared with diffusive electrical stimulation (34). Although electrical stimulation combined with other treatments such as rehabilitation shows therapeutic effect on treating SCI (12, 16), our study shows that optogenetic neuromodulation alone is sufficient to promote neural reorganization both in the cortex and spinal cord, leading to functional recovery.

An important observation in the present study is that selective activation of the motor cortex is sufficient to establish an alternative corticospinal motor pathway leading to forelimb functional recovery following a C5-RH, mimicking the human Brown-Séquard syndrome. Using *Thy1-ChR2* transgenic mice, we show that selective activation of the excitatory neurons by optogenetic stimulation induced the intact CST axons on the contralesional side to sprout and make synaptic connections with ipsilesional spinal neurons below the level of injury. This stimulation-mediated neural reorganization itself, without any other combination, led to both general and skilled forelimb functional improvements in a very severe cervical RH model, which destroys not only the ipsilesional CST but also all other descending and ascending pathways on the ipsilesional side.

Reorganization of neural circuitry occurs after SCI, and it is important to understand such remodeling of circuitry on regulating specific functional recovery. In the current study, we determined that the contralesional intact CST pathway played a key role in bringing back the lost function of the impaired ipsilesional forelimb. After a complete C5-RH, all rostral descending supraspinal and propriospinal tracts (as well as ascending sensory tracts), were transected on the ipsilesional side, leading to the loss of skilled motor control over the forearm and digits on the ipsilesional side. Since, after a C5-RH, severed CST axons fail to regenerate spontaneously, the functional deficit of the ipsilesional forearm and digits is permanent. However, we demonstrated that functional improvement of the impaired forelimb was achievable after the optogenetic stimulation and that such improvement was based on the reconstruction of a newly formed, alternative CST pathway involving the sprouting of intact CST axons from the contralesional side to the ipsilesional side and their synaptic connection with the ipsilesional motor system that control the forelimb function. Construction of such an alternative corticospinal motor pathway, promoted by optogenetic stimulation, led to prominent recovery of the impaired forelimb function following a unilateral C5-RH. Interestingly, in sham-operated mice with no SCI, optogenetic stimulation also increased axonal sprouting of the intact CST on the contralesional hemicord. This optogenetic stimulation that modulated intact CST sprouting, however, did not interrupt the normal motor function in these animals, indicating that optogenetic stimulation does not interrupt the existing motor functional neural circuitry. Since we aimed to study motor function after SCI, the sensory function, which has also been severely damaged in this injury model, was not explored. Chronic neuropathic pain is found to be related to the neural plasticity after SCI (35). For example, the anterior cingulate cortex (ACC) is important for acute pain perception as well as the development of chronic neuropathic pain (36). Because neuropathic pain following SCI is often resistant to treatment clinically, there is also an urgent need to understand the connection between neuropathic pain and circuit plasticity. However, this is beyond the scope of this study, which is focused on the motor functional improvement.

Selective activation of the contralesional spinal CST motor circuits promotes both skilled and locomotive forelimb motor recovery after the C5-RH. Forelimb function is important for the quality of life in SCI patients. However, it has been less studied after a severe cervical SCI. Like the human Brown-Séquard syndrome, mice with cervical RH showed severe functional deficits in locomotion (37) and skilled movement. Following the cervical lateral hemisection, the main descending pathways, including cortico-, rubro-, vestibulo-, and reticulo-spinal tracts and the modulatory serotonergic, dopaminergic, and noradrenergic fiber systems were disconnected by the lesion, leading to poor forelimb functional recovery. Impressively, we found that optogenetic stimulation promoted a significantly greater general locomotor recovery as compared with the nonstimulation group. Optogenetic stimulation also significantly improved skilled function, assessed by pellet retrieval parameters with EWMN measurements, and grid walking analysis as compared with the nonstimulation group. Although the dexterous hand function, which is critical for quality of life in humans, improved in the neuromodulation group, the full pellet retrieve recovery was not achieved. Further improvement of the forelimb function may require a combinatorial treatment including promoting the damaged CST axons to regenerate on the ipsilesional side.

Although the CST is required for forelimb function, it is not the only pathway modulating the locomotive and skilled movement of the forelimbs. Other descending pathways are also involved in motor control. For example, the medullary reticular nucleus (MdV) located in the brain stem is known to receive cortical input and descend its axons through the ipsilateral dorsolateral funiculus to the spinal cord. The MdV axons form monosynaptic connections with both excitatory and inhibitory spinal interneurons at the cervical level, which is of special significance for skilled function (38). It is possible that the MdV may indirectly mediate the CST on forelimb function. Additionally, other regions of the brain stem centers may undergo various degree of remodeling and play a role in forelimb function following SCI. For example, DBS of the midbrain locomotor region was shown to improve hindlimb functional recovery after SCI (13). Previously we showed that the descending propriospinal tract (dPST) plays a central role in NT-3–mediated hindlimb functional recovery (39). Thus, the subcortical and intraspinal neurons, which receive corticospinal projections, could also play a role in regulating the optogenetic-modulated functional recoveries. These observations lead us to believe that a significant increase of compensatory CST axonal remodeling, an orchestra with the plasticity of other pathways, promoted multiple types of behavioral recoveries that are either CST-specific or CST-associated.

Optogenetic-stimulated mice moved longer distances than the nonstimulated mice in the SmartCage analysis of this study. This so-called “general exercise,” triggered by optogenetic stimulation, could be another reason for the improvement in general locomotor recovery. However, such an exercise could possibly make skilled paw reaching function worse. This “one behavior training makes another behavior worse” effect has been reported previously (40), indicating different patterns of newly formed neural circuitry compete for specific functional control. Thus, to target a specific motor function, a combination of task-specific trainings with optogenetic stimulation would be a future direction. An earlier study showed that, when combining exercise training with optogenetic stimulation of the CST, improved motor function can be found in a stroke model (41). With the development of brain-machine interface, decoding motor output has provided knowledge to promise new therapeutics in treating SCI (42–44).

Finally, we demonstrated a long-lasting therapeutic effect of optogenetic stimulation on CST axonal sprouting and recovery of forelimb function. This could be due to our intense stimulation strategy. Unlike other short-term (several minutes) optogenetic stimulations (45, 46), we chose an aggressive and repetitive stimulation strategy that stimulate animals for 6 hours per day during a consecutive 7-day period, similar to the earlier electrical paradigm used for experimental pyramidotomy (17). Interestingly, activation of cortical pyramidal neurons via optogenetic stimulation showed increased expression of BDNF, a critical neural plasticity modulator, not only in the cortex, but also in the injured spinal cord, suggesting this remodeling occurred throughout the entire CST system, agreeing with the results of neural reorganization in both the cortex and the spinal cord.

## Methods

Detailed methods are provided in the Supplemental material.

### Study approval.

All experimental procedures including surgical interventions, behavior and electrophysiological assessments, and postoperative animal care were performed in accordance with the “Guide for the Care and Use of Laboratory Animals” (National Research Council) and approved by the Institutional Animal Care and Use Committee of the Indiana University School of Medicine.

### Statistical Analysis.

Statistical analysis was performed using 2-tailed Student’s t-tests, 2-way ANOVA with group and time as variables, and Tukey’s multiple comparisons test using Graphpad Prism 7.0 between all groups in each experiment. Statistical significance was accepted with *P* < 0.05. Data are expressed as mean ± SEM. Linear regression models were used to estimate the effect of optogenetic stimulation on behavioral tests and to determine the magnitude of stimulation effect accounted for by axonal sprouting, using percent changes in R2 for stimulation with the adjustment of axonal length in the models. JMP analysis 11 was used for group comparison in the brain mapping study.

## Author contributions

WW designed and performed the experiment, analyzed the data, and wrote the manuscript; JDO performed imaging and data analysis; TN, XP, and XJ performed brain mapping experiments; NKL performed Western blot analysis; YZ and CBS built up the LISA laceration system; XH and SG performed statistical correlation analysis; YL performed optical stimulation; WQ, QH, and XW performed behavior tests; and XMX designed the experiment and wrote the manuscript.

## Supplementary Material

Supplemental data

Supplemental video 1

## Version 1 05/12/2022

In-Press Preview

## Version 2 06/22/2022

Electronic publication
