# Supplementary material for: Transhemispheric cortex remodeling promotes forelimb recovery after spinal cord injury
Source: JCI Insight. 2022 Jun 22;7(12):e158150. doi: 10.1172/jci.insight.158150 (PMC9309060; doi:10.1172/jci.insight.158150)
Supplement: Supplemental data [file jciinsight-7-158150-s213.pdf]

Supplementary Information

Methods

Animals. A total of 148 female Thy1-Channelrhdopsin 2(ChR2) transgenic mice (line 9, stock number 7615, 8 weeks, Jackson lab), housed in groups of 2 to 4 under a constant 12 h dark/light cycle, with food and water supplied, were used for this study. We chose female mice over male mice because no difference was found in functional and histological outcomes after a contusive SCI in age-matched rodents (rats) in our recent study (1).

Cervical 5 right hemisection. C5 right hemisection (C5-RH). Mice were anesthetized with intraperitoneal (i.p.) injection of a ketamine/xylazine/acepromazine mixture (100mg/3.3mg/1.67mg/kg). To minimize the movement of the spinal column at the injury site and precisely produce a right-sided hemisection, a spine stabilizer was used as described in our previous studies (2). A 1 cm midline incision was performed and the spinal cord between C4-5 vertebrae was exposed after removing the ligaments. The dura was punctured with a needle (30G x ½, 0.3 mm x 13 mm) in the midline and cut to the lateral edge using a pair of superfine iridectomy scissors. The right hemicord was then lacerated using a VibraKnife attached to the Louisville Injury System Apparatus (LISA), which can achieve 0.01mm cutting accuracy(3). To protect the left spinal cord and confirm all the right spinal cord had been cut thoroughly, we used a modified needle (30G x 1/2) to insert into the midline of the spinal cord (**Fig. S1B**). A thin blade (width: 1 mm) then cut the right spinal cord tissue along with the needle as shown in **Fig. S1B**. This approach allows the transection of all passing axonal tracts at the site of C5-RH.

Optogenetic stimulation. After the C5-RH, under the ketamine/xylazine/acepromazine anesthetization, the scalp directly above the sagittal suture was cut to expose the skull, and the area was dried using cotton balls. After a layer of cyanoacrylate glue was applied, a high-intensity blue light-emitting diode (LED, ~470 nm peak wavelength) (LED Lighting Color XLamp®XP•E Blue 475nm, DigiKey) was then glued on the skull above the right (ipsilesional) motor cortex. The LED is controlled by a miniature circuit that generates a burst of 3 pulses at 33 Hz once every 3 seconds. In the stimulation group, stimulation was given for a total of 6 hours (3 hours x 2 time per day with 1-hour interval) for a total of 7 days. For the non-stimulation group, the LED was installed on the skull the same way as the stimulation group but with the light off.

Corpus callosotomy. Mice were anesthetized as described above. The skull above the sensorimotor cortex was exposed and the periosteum was cleaned. A 4 mm long slit was drilled in the skull, at 0.5 mm lateral to the midline and parallel to the sensorimotor cortex. An "L"-shaped bent 34-gauge needle was inserted into the midline between 2 cortical hemispheres and lowered vertically 1.5 mm to cut the corpus callosum. Optogenetic contralesional and ipsilesional recordings were conducted at 1 day after corpus callosotomy.

Behavioral testing. Forelimb skilled function assessments. The forelimb skilled function assessments include the single-pellet retrieval test and grid walking tests, as described below:

Single-pellet retrieval test: The single-pellet retrieval test has been used to test the precise and coordinated motor movements, so-called dexterous function, of the forelimb. This test was performed and modified according to our previously published work (4). Briefly, mice were trained to use their left and right forelimb to retrieve food to test their ipsilesional and contralesional forelimb function respectively (**Figure 5A & Figure S2A**). For each side, the mouse performed the test for a total of 15 minutes or until they brought 20 pellets into the chamber. Recordings were done by using two Sony camcorders for obtaining both front view and side view. After baseline recording, a C5-RH was performed as described above. Mice then continued the test at every 2 weeks as did in the baseline recording. During the entire testing period, mice weights were tracked. Any mouse whose weight dropped more than 10% of the baseline body weight was excluded from the study. After testing, videos were analyzed. Four parameters were used including fail to touch, touch, fail to retrieve, and retrieve. "Fail to touch" means that the mouse tries to grab the pellet but missed the it; "Touch" is defined as the mouse has contacted the pellet but fails to grasp and take it into the chamber; "Fail to retrieve" is defined as that the mouse has grasped the food but failed to take it to the chamber; "Retrieve" is defined as when the mouse can grab the pellet, put it into the mouse and eat it. (**Fig. 5C**). These attempts were counted for each animal on either side. The test time for each side was recorded to measure the rate of each parameter. Ratio of each parameter was calculated by dividing the number of attempts of a particular parameter by the sum of all attempts. Statistical analysis was done using two-way ANOVA with Tukey's post hoc test. The Eshkol-Wachmann Movement Notation (EWMN) score was performed to further analyze specific movement(s) during the whole reaching and retrieving processes (5, 6). Briefly,

3-point scale was utilized to evaluate each movement: 0 for normal, 1 for incomplete or moderate impairment, 2 for absence or severe impairment.

Grid walking test: The grid walking test is a sensitive test for evaluating the sensorimotor coordination of the fore- and hindlimbs and the descending motor control of the limb motor pathways. This test was performed following our established protocol (4). Briefly, animals were allowed to walk on a wire grid (grid size 10 x 10 mm2) for 3 minutes (**Fig. 6A**), and foot drops of the left and right forelimbs were counted separately. A percentage of forepaw drops below the grid plane was calculated at 3 d before injury (as a baseline), and at 2, 4, 6, 8 weeks after the right hemisection.

General locomotor function was examined using cylinder, rotarod, and SmartCage locomotor tests as described below:

Rotarod test: The rotarod test was used to assess motor coordination in rodents according to our previously published protocol (4). Briefly, mice were placed on a rotating rod (diameter 30 mm) of a Rotarod device (IITC Life Science) that accelerated from 0-30 rpm with a 90-second period (**Fig. 6A**). The total testing duration per each mouse was 120 seconds, and the times when the mice fell from the rotating bar was recorded for each mouse. The rotarod test was performed before the injury, and 2, 4, 6, and 8 weeks after the C5-RH (**Fig.6C**).

Cylinder test: The cylinder test was used for testing the forelimb asymmetry as we described before (4). Mice have a natural behavior of exploring vertical surfaces by rearing up on the hindlimbs and exploring the surface with the front paws. The number of times a mouse assumed a clear upright position in a beaker (as a cylinder, **Fig. 6A**) and independently places its left, right, or both forepaws against the glass wall was recorded using a video camera. The percentage of left, right or both forepaw usage was calculated to indicate each forepaw's movement before SCI (baseline), and at 2, 4, 6, and 8 weeks after the right hemisection (**Fig. 6D**).

SmartCage test: The SmartCage system is an automatic, non-invasive rodent behavior monitoring homecage system used to examine mouse activities. This test was conducted following our previously published work (7). Briefly, we placed a single mouse in the SmartCage system (AfaSci, Inc., San Francisco, CA) under a dark environment to encourage mice to move freely for 1 hour. Activity of the mice was recorded and analyzed using CageScore software (AfaSci, Inc.).

Mice were divided into 2 groups: right hemisection + non-stimulation (RH + non-Stim) (n=8) and right hemisection + stimulation (RH + Stim) (n=12). We tested moving distance and velocity before the injury (baseline) and at 8 weeks postinjury, as indicators for functional improvements.

Biotinylated dextran amine (BDA) anterograde tracing. BDA (10%, 10,000 MW, Invitrogen) was used as an anterograde tracer to label the CST axons at 6 (short-term) and 10 (long-term) weeks postinjury. For the short-term study, 9 mice were divided into 2 groups: RH + non-Stim (n=4) and RH + Stim (n=5). For the long-term study, 24 mice were divided into 4 groups: Sham + non-Stim (n=4), Sham + Stim (n=5), RH + non-Stim (n=8), RH + Stim (n=9). For each mouse, 5 injections (0.5 μl/injection) of BDA were made into the ipsilesional sensorimotor cortex to track the intact CST on the contralesional side. The injection was made via a glass micropipette attached to a 10 μl syringe connected to the Stoelting motorized integrated stereotaxic injector system (Stoelting, Wood Dale, IL) according to the following coordinates (in mm): anteroposterior (AP)/mediolateral (ML): 2/1.5, 1.25/1.5, 0.5/1.5, -0.25/1.0, -1/1.0, at depths of 0.7 mm. Mice were allowed to survive for an additional 2 weeks before perfusion and tissue harvest for BDA visualization of labeled CST axons.

Immunofluorescence staining. Immunostaining was conducted following our previous standard protocol (4, 8). Mice were given a lethal dose of anesthesia and transcardially perfused with phosphate buffered saline (PBS) and 4% paraformaldehyde (PFA). Spinal cords were isolated and postfixed in the 4% PFA solution overnight at 4°C. Tissues were dehydrated via increasing concentrations of sucrose (up to 30%). Then the spinal cords were sectioned either transversely or horizontally at 25 μm-thick using a cryostat. The lesion was defined by immunofluorescent labeling of glia fibrillary acidic protein (GFAP; 1:400; AB5804, Chemicon, Temecula, CA). BDA–labeled CST axons were stained using avidin-biotin peroxidase incubation, followed by standard TSA staining protocol (SAT700, PerkinElmer, Waltham, MA).

CST axonal counting and quantification. All axon counting and quantification was conducted in a double-blinded manner. To correct for variations in BDA labeling, number of dorsal CST axons at cervical 2 (C2) was counted for normalization in each case. In all groups, digital images stained for BDA-labelled CST axons were taken by Neurolucida system (MBF BIOSCIENCE, Williston, VT). Each CST axon was manually drawn at both ipsilesional and contralesional side. The outcome measurement was presented as axon length index (ALI) and axon number index (ANI).

ALI represents the total length of axons on one side of the spinal cord normalized by the number of axons in the C2 dorsal CST. Six sections were measured for each mouse, including 3 sections above and 3 sections below the injury. ANI is the axon number at different distances from the midline normalized by the total number of axons in the C2 dorsal CST. To quantify the ANI, a grid with 100 μm spaces was created using Adobe Photoshop CS (Adobe Inc. San Jose, CA). This grid was then copied into PowerPoint along with a print screen of the Neurolucida image with drawn axons. The grid was then aligned on one side of the spinal cord by placing the left or right end of the vertical bars from the grid on the central canal. The grid was copied and pasted on this image and reflected it to place on the other side of the spinal cord. PowerPoint slides were then exported as Tiff files and axons within each 100μm wide boxes were counted using Image J Fiji cell counter function (NIH). Numbers of axons between each box were averaged from at least 3 sections both above and below the injury.

Synapse quantification. Tissue sections were immunostained for MAP2 and synaptophysin. Sections were blocked with 10% BSA + 1% Triton X-100 for 1 hour at room temperature on a shaker. Blocking solution was then aspirated. A solution containing 1:500 rabbit anti-MAP2 (AB5622, Millipore Sigma, St. Louis, MO) and 1:200 mouse anti-synaptophysin (MAB329, Millipore Sigma, St. Louis, MO) primary antibodies and 5% BSA/0.5% Triton was added to the slides and kept overnight (about 15 hours) at 4°C in a shaker rotating at minimal speed. Cells were then washed 3 times using PBS. A solution containing 1:1,000 Alexa 405 goat anti-rabbit (AB175652, Abcam, Chemicon, Temecula, CA) and 1:200 Cy5 donkey anti-mouse (715-175-151, Jackson ImmunoResearch, West Grove, PA) secondary antibodies in PBS were then added and tissues were kept for 2 hours at room temperature on a shaker. Sections were then washed 3 times with PBS and sealed using 1mm rectangular cover slips and Fluoromount solution. Slides were kept in dark overnight at room temperature to allow the Fluoromount to dry. Images were taken using an Olympus confocal microscope (FV1200, Shinjuku, Tokyo, Japan) and FV10-ASW 4.2 capturing software. Consistent images for each channel (TRITC for BDA; Alexa 405 for MAP2; CY5 for synaptophysin) in each tissue section were taken. A step-size of 1μm was used for 20X images and 0.5μm for 60X images. Images were exported as merged channels using Fiji Image J (NIH), and Z-stack images were combined using Z project function and the Max Intensity projection type. Triple labelling was then manually counted with the aid of a cell counter to keep

track of counts. Synapse formation was detected by white color which corresponded to triple labelling. Total images taken for control were 9 (3 images per group, 3 groups) at ~750-2250 μm caudal to the injury. Total images for treatment group were 12 (3 images per group, 4 groups) and at ~750-2250 μm caudal to the injury. Statistical analysis was performed using two-tailed t-test.

Optogenetic cortical mapping with EMG recording. Mice were anesthetized with a mixture of ketamine (83 mg/ml) and xylazine (8 mg/ml) and fixed in a stereotaxic frame. Under light anesthesia, animals were responsive to tail pinch and this anesthetic condition was maintained throughout the course of experiment. The optogenetic mapping set up used was similar to the one described by Ayling O.G. et al. (9). A 470-nm laser light at 5 millisecond (ms) duration was generated at 10 Hz pulses and applied onto the cortex. Each mapping region consisted of a rectangular 5 x 6 grid with 300 μm spacing between the grid (1.5-by-1.8 mm) shown in **Figure S1A**, which was generated using Laser Hop modular software (Stanford University, Stanford, CA). Laser pulse was applied randomly onto each spot with an inter-pulse delay of 10 ms until all spots were covered. Each cortical hemisphere consisted of 6 mapping regions with respect to the bregma with total dimension of 4500-by-3600 um **(Supplementary Fig. 3A)**. Forelimb electromyograms (EMGs) were recorded from anterior biceps brachii using electrode similar to those described by Harrison et al. (9, 10). EMG signals were amplified using DP-304 Differential Amplifier (Warner Instrument Corp, Hamden, CT), processed with Digidata signal processor (Molecular Devices), and output via pClamp 4.1 (pClamp 10 software, Digidata 1440 interface; Molecular Devices). For each animal, each region was mapped repeatedly 3 times and spots with responses 2 out of 3 times or more were counted as active spots. One out of 3 responses or no response were scored as unresponsive (11). Laser stimulation was set at 10 ms delay from the start of EMG recording. Response latency was calculated as the period between the start of laser stimulus and the time of the emergent EMG signals. All response latencies were measured from the maximum response EMG on the map of each animal at each time point (baseline and 8 weeks).

EMG responses were analyzed using DetectorAnalysis (Stanford University, Stanford, CA) to obtain peak amplitudes. Reported amplitude for each stimulated spot was the average amplitude of three repetitions. Spot numbers were obtained by counting only stimulated spots that produce EMG response. Maximum EMG amplitudes, with respect to rostral-to-caudal and medial-to-lateral positions, were determined from EMG amplitudes for all active spots at each position along both

directions. Maximum EMG amplitudes were calculated for each animal at each time point. Change in maximum amplitude was determine by normalizing the amplitude of each time point to baseline. Data shown are average changes in maximum amplitudes after pooling together all animals at each respective time point. Motor cortical activity maps were constructed with OriginPro9.1 (OriginLab, Northampton, MA). Amplitude calculation and normalization were done with Microsoft Excel. Statistical analysis was done with JMP Analysis 11 (SAS Institute Inc. 2013. Cary, NC) (11).

Western blotting. Western blot analysis was performed according to our protocol as described previously (12). Briefly, an equal protein concentration from each sample was loaded onto polyacrylamide gel, separated by SDS-PAGE, and transferred to a nitrocellulose membrane by electrophoresis. The membrane was blocked in Odyssey blocking buffer for 1 hour at room temperature. The primary antibodies were added to the membrane and incubated for overnight at 4°C. The primary antibodies included rabbit anti-BDNF (1:1000, abcam, Cambridge, MA) antibody, and mouse anti-β-tubulin antibody (1:1000, Sigma, St. Louis, MO). The membrane was washed 4 times for 5 minutes with PBS-T (PBS + 0.1% Tween-20) at room temperature, incubated with a secondary IRDye 800 goat anti-rabbit (1:5000, Rockland, Gilbertsville, PA) or Alexa Fluor 680 goat anti-mouse (1:5000, Invitrogen, Grand Island, NY) for 1 h, then washed 3 times for 5 minutes with PBS-T and twice for 5 minutes with PBS. The Western blot was imaged and quantified using a Li-Cor Odyssey Infrared Imaging system (LI-COR Biosciences, Lincoln, NE) according to the manufacturer's instruction. For the negative control, the primary antibody was omitted.

Statistical Analysis. Statistical analysis was performed using two-tailed Student's t-tests, two-way ANOVA with group and time as variables, and Tukey's multiple comparisons test using Graphpad Prism 7.0 (SanDiego, CA) between all groups in each experiment. Statistical significance was accepted with $p<0.05$. All data are expressed as mean ± s.e.m. Linear regression models were used to estimate the effect of optogenetic stimulation on behavioral test and to determine the magnitude of stimulation effect accounted for by axonal sprouting using percent changes in R2 for stimulation with the adjustment of axonal length in the models. JMP analysis 11 (SAS Institute Inc. 2013. Cary, NC) was used for group comparison in brain mapping study.

Supplementary Figures and Legends

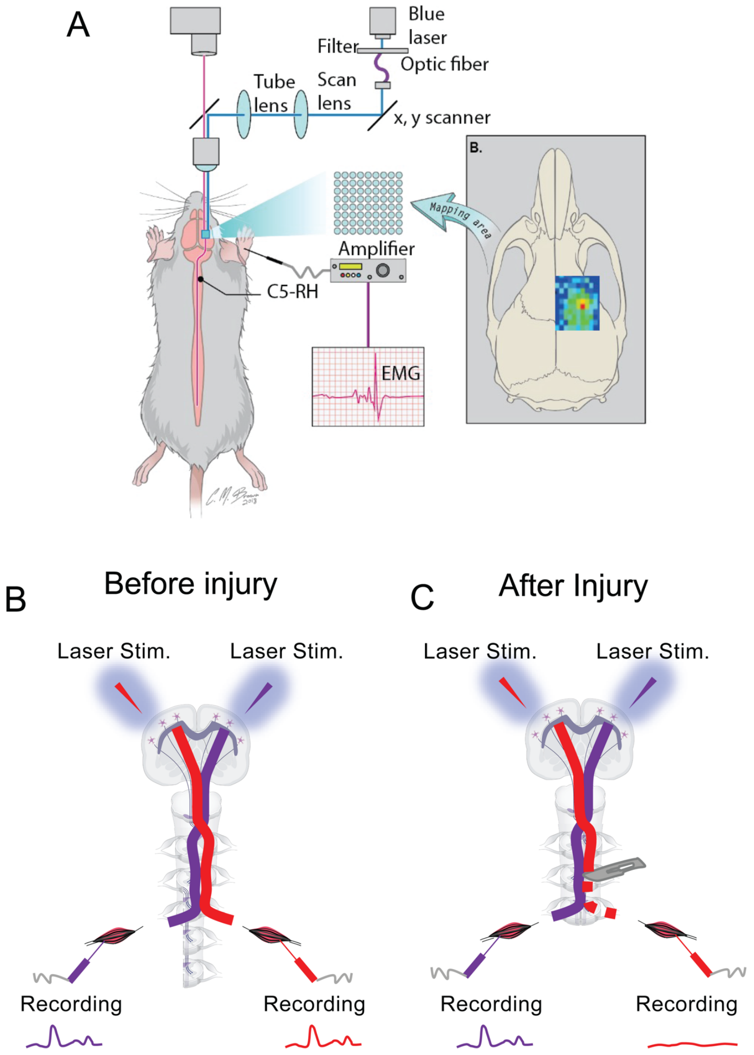

Supplementary Fig 1. Optogenetic mapping confirms the ipsilesional CST disruption after the C5-RH. (A) Schematic drawing of the cortical optogenetic mapping setup. (**B**) Baseline recording before the injury. Optogenetic stimulation with a laser beam on one side of the motor cortex elicits an electrical recording of the anterior biceps on the opposite side, shown in red and purple colors, respectively. This recording can be presented as a cortical map, indicating that the intact CST transmits electrical signal from one side of the motor cortex to the opposite side of the forearm muscle. (**C**) After the C5-RH, stimulation of left motor cortex does not elicit the right forelimb muscle response, indicating that the CST on the right side (red dashed line) was

completely destroyed. In contrast, stimulation of the right motor cortex elicits the left forelimb muscle response, indicating that the CST on the left side (purple) remains intact after the C5-RH.

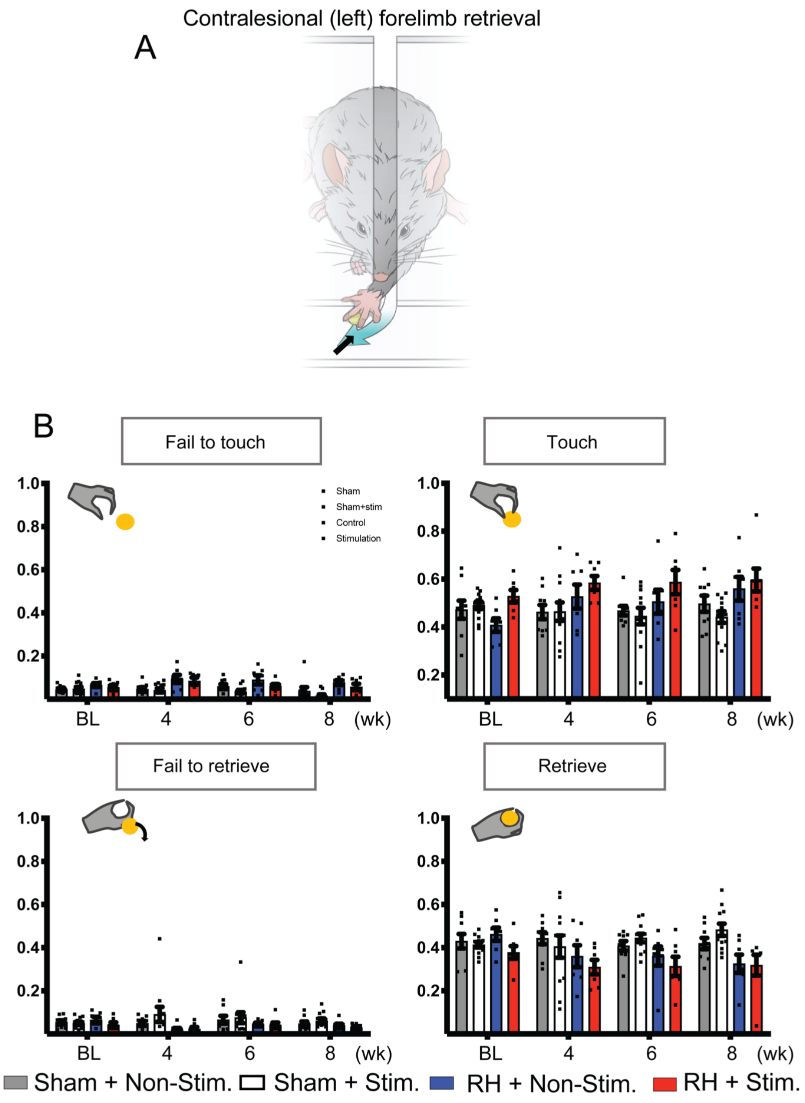

Supplementary Fig 2. Transcranial Optogenetic stimulation does not affect contralesional forelimb pellet retrieval. (**A**) Schematic drawing shows the contralesional (left) forelimb pellet retrieval. (**B**) Optogenetic stimulation did not affect the contralesional forelimb function as compared to the non-stimulation group in both the sham and C5-RH groups, indicating that

transcranial optogenetic stimulation does not affect the forelimb pellet retrieval function on the contralesional (left) side where the CST remains intact. Data were presented as means ± s.e.m; n = 7-12; two-way ANOVA followed by Tukey's multiple comparisons test.

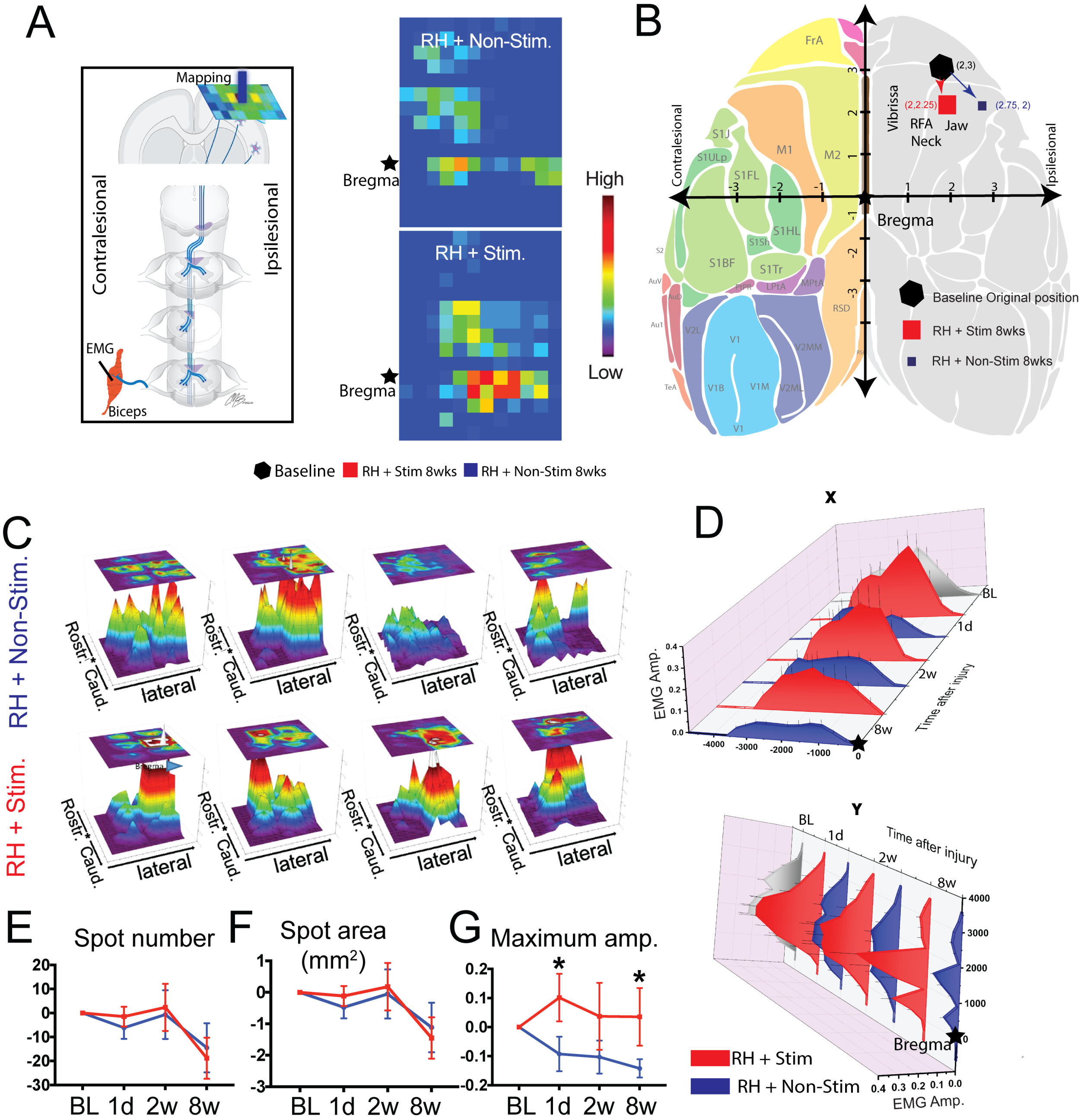

Supplementary Fig 3. Optogenetic stimulation increases the spared CST activity on the contralesional side after the C5-RH. (A) Left, schematic drawing illustrates how the mapping

conducted. Right, representative heat map shows the increased response in optogenetic stimulation group at 8 weeks after the C5-RH as compared to the non-stimulation group. **(B)**Cortical map reorganization in both the stimulation and non-stimulation groups. Black hexagon, the dot with maximum response in left biceps before C5-RH. Blue square, the dot with maximum response in left biceps at 8 weeks after C5-RH with no optogenetic stimulation. Red square, the dot with maximum response in left biceps at 8 week after C5-RH with optogenetic stimulation. Size of the marker is proportional to the percentage of mice with EMG amplitude in biceps. **(C)** Maximum amplitude projection on x- and y- axes. (**D, E, F**) Quantitative comparison of spot number, response area, and amplitude between the stimulated and non-stimulated groups. $n = 6$ per group. Data are presented as means ± s.e.m; statistical evaluation was carried out with two-way ANOVA repeated measure followed by Tukey's multiple comparisons test; asterisks indicate significances: $*p < 0.05$.

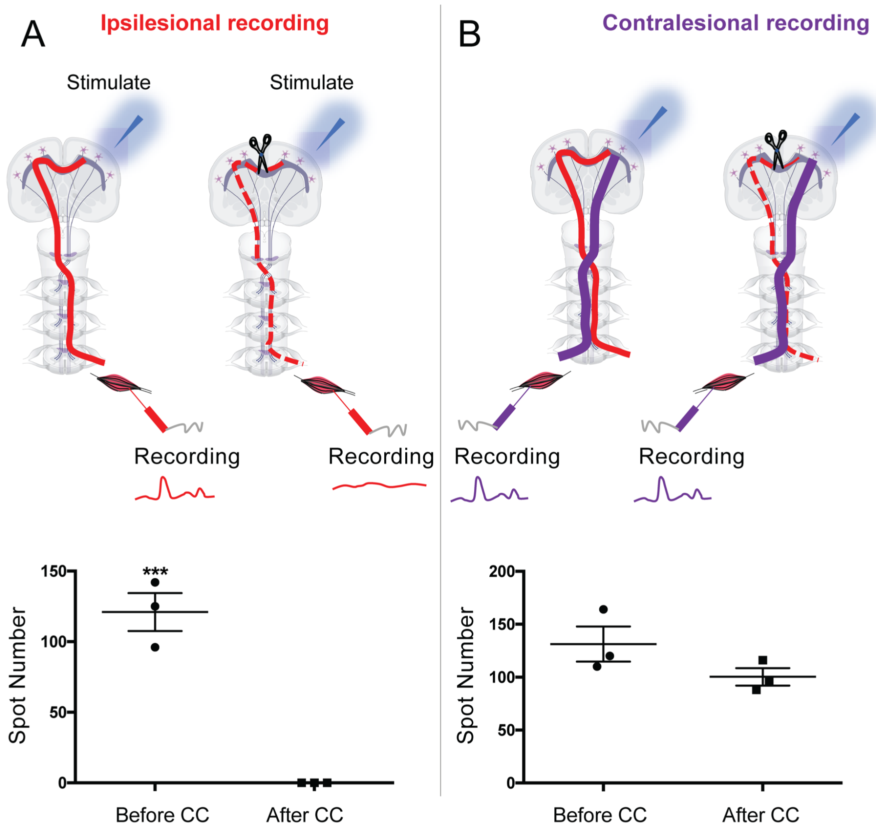

Supplementary Fig 4. Corpus callosotomy reveals the presence of cortico-cortical motor connectivity through the corpus callosum. (A) In the intact spinal cord, stimulation of one side

of the motor cortex can elicit biceps recordings on the same side, an unexpected observation. Such recording was disrupted after corpus callosotomy, indicating that signals from one side of the motor cortex can be transmitter to the other side of the motor cortex to control motor activity on the same side of cortical stimulation. **(B)** When stimulating one side of the cortex and recording the contralateral side of the muscle, no change in electrical signals was found after corpus callosotomy, indicating that corpus callosotomy does not affect the signals transmitted through the intact CST on the contralateral side. Data are presented as means ± s.e.m; n=3 animals were tested, two-tailed paired Student's t-test. Asterisks indicate significance ***p<0.001.

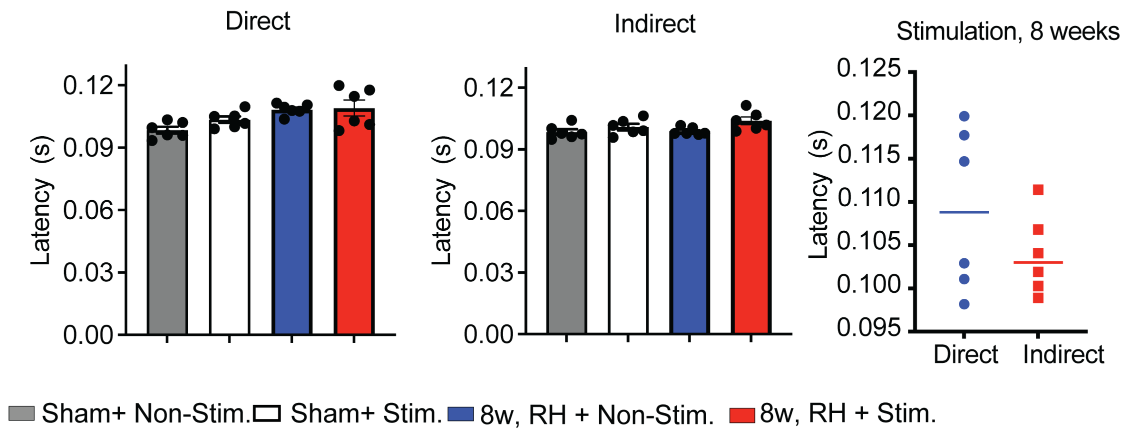

Supplementary Fig 5. The latency comparison from direct and indirect recording at different timepoints. No significant difference was found in direct and indirect recording among sham + non-stimulation, sham + stimulation, RH + non-stimulation, and RH + stimulation groups. The latency was analyzed at baseline (sham) and 8 weeks after right hemisection.

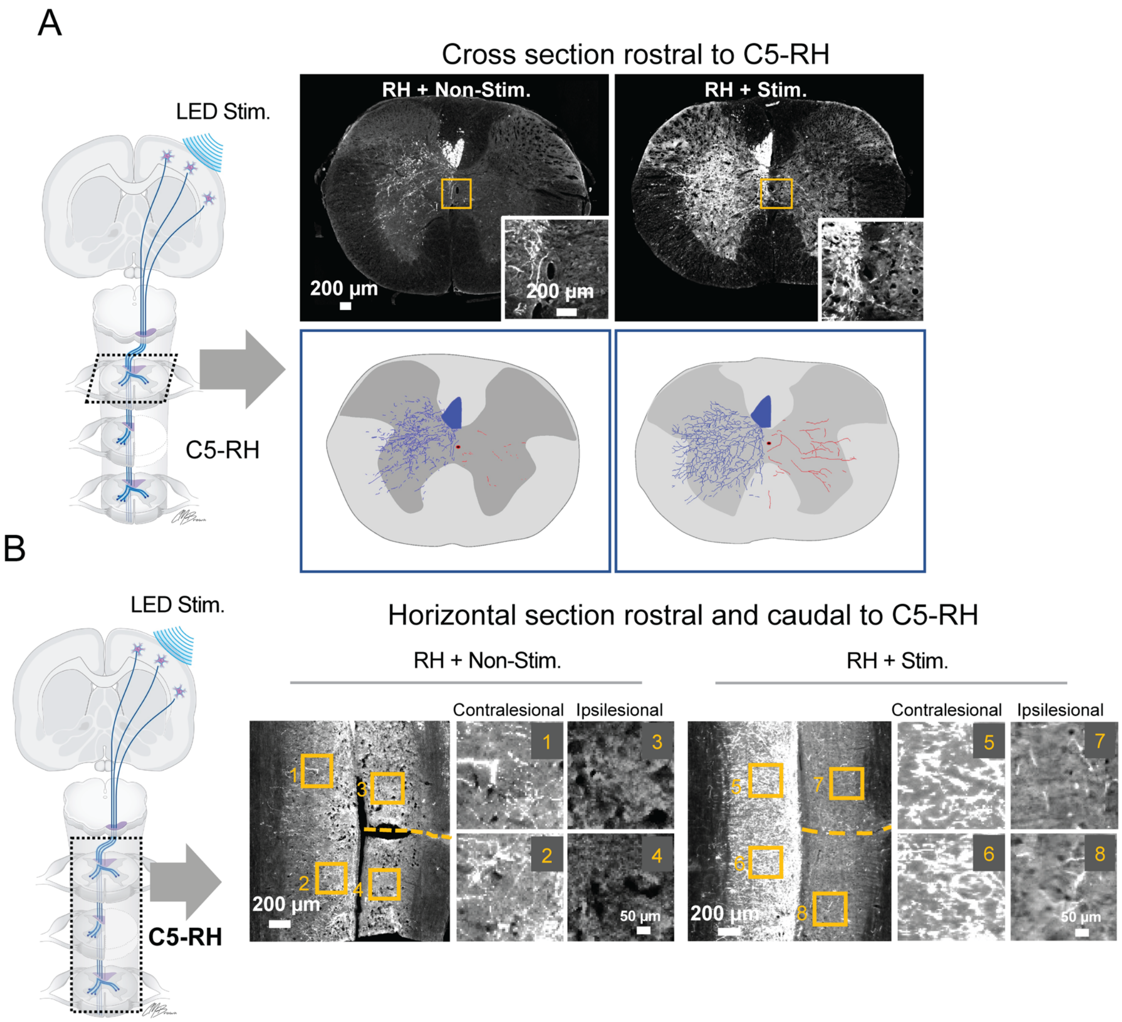

Supplementary Fig 6. Transcranial Optogenetic stimulation promotes the intact CST axons to sprout from the contralesional side to the ipsilesional side at levels above and below the C5-RH. (**A**) (Left) Schematic drawing shows a cross section taken from the level above the C5-RH. (Right) Representative spinal cord cross sections showed enhanced CST axonal sprouting across the midline (red axons in the neurolucida drawing) only after the optogenetic stimulation at 6 weeks after a C5-RH, as compared to the lack of sprouting in the non-stimulation group. Higher magnification of boxed areas further demonstrates the presence or absence of crossed CST axon terminals in the ipsilesional gray matter. Representative Neurolucida drawings show the uncrossed (blue) and crossed (red) CST axons in non-stimulated and stimulated conditions. (**B**) Left: Schematic drawing shows a horizontal section taken from above, at, and below the C5-RH. Right,

Top: Optogenetic stimulation enhanced CST axonal sprouting across the midline to the ipsilesional side at levels both rostral and caudal to a C5-RH (dashed line). Right, Bottom: High magnifications of boxed areas matched the corresponding numbers indicated in the (Right, Top).

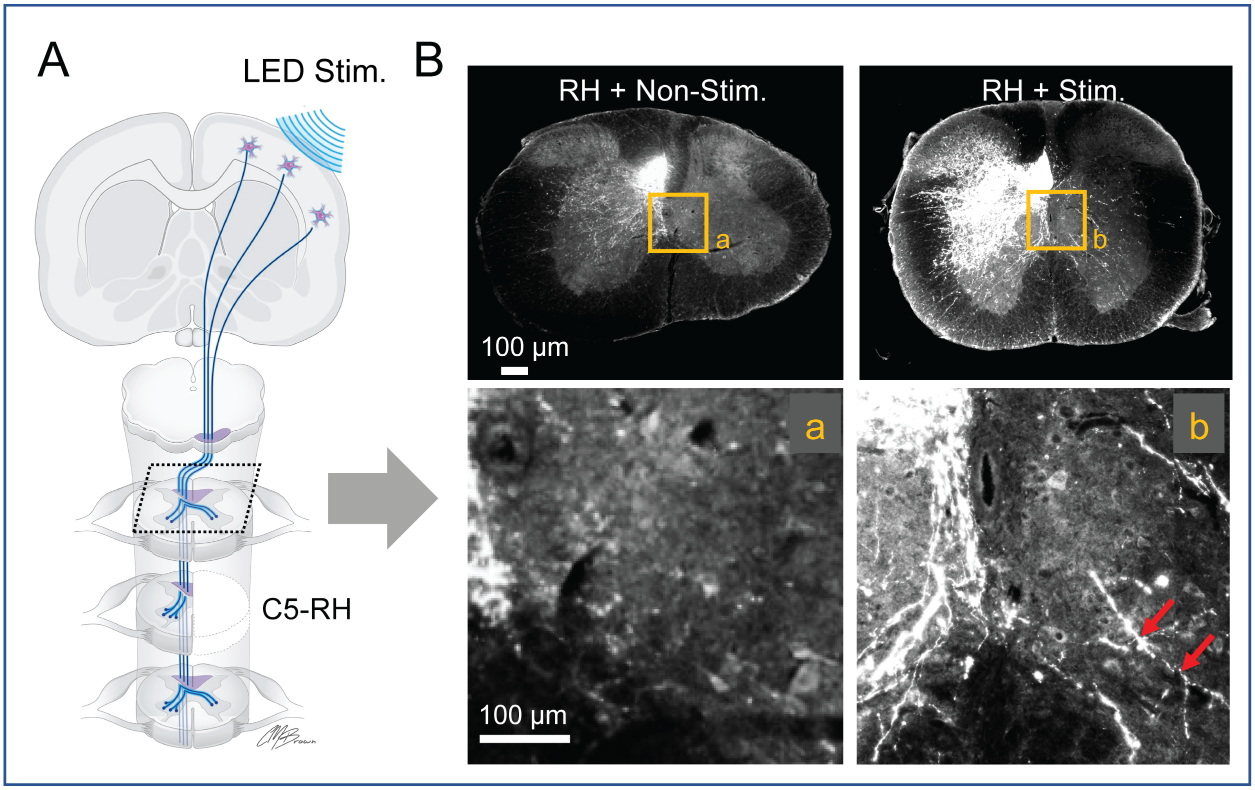

Supplementary Fig 7. Transcranial optogenetic stimulation promotes the intact CST axons to sprout from the contralesional side to the ipsilesional side at 10 weeks after the C5-RH. (**A**) Schematic drawing shows where the sections were taken. (**B)** Representative cross sections show BDA-labeled CST main tract and axon branching into the spinal gray matter. Robust CST axonal sprouting across the midline was only found in the group that received optogenetic stimulation as compared to the non-stimulation group. High magnification of boxed areas further demonstrates the presence (red arrows) or absence of crossed CST terminals in the ipsilesional hemicord.

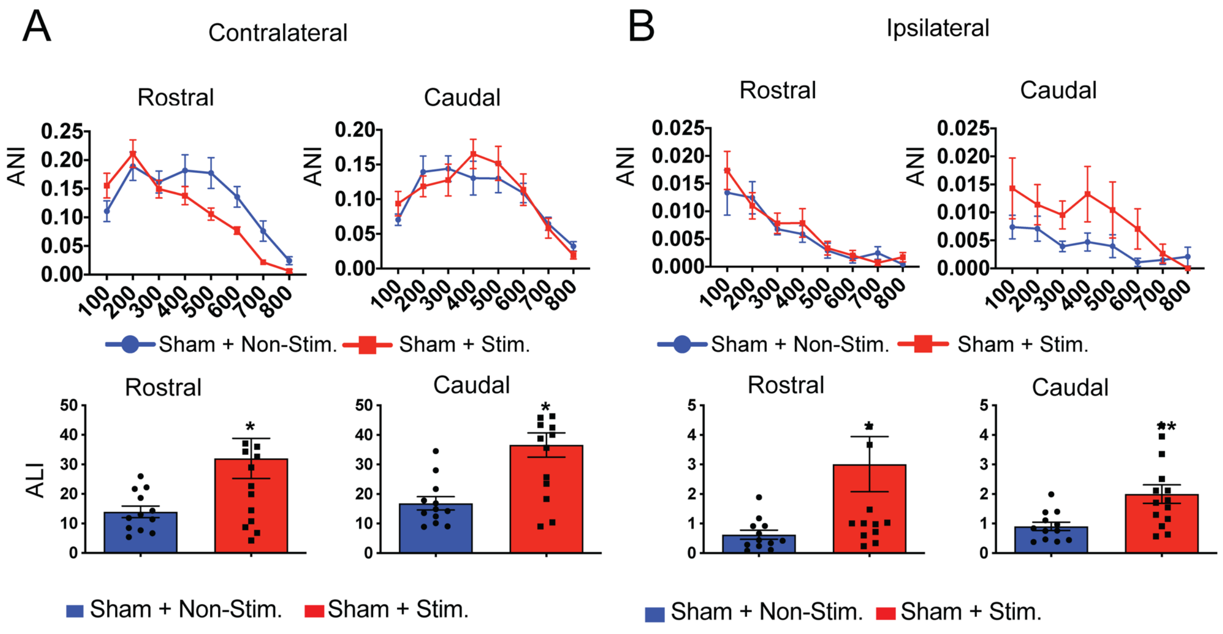

Supplementary Fig 8. Optogenetic stimulation increases the CST axon length, but not the number of branches, in sham groups. (A) Quantitative comparison of CST axons between the optogenetic stimulation and non-stimulation groups was performed. **(B)** Quantitative comparison of CST axons between the optogenetic stimulation and non-stimulation groups was performed. ANI - axon number index; ALI – axon length index. Sham + Non–Stim (n=4); Sham + Stim (n=5). Data are presented as means ± s.e.m; statistical evaluation was carried out with two-way ANOVA repeated measure followed by Tukey's multiple comparisons test; asterisks indicate significance: *p<0.05, **p<0.01.

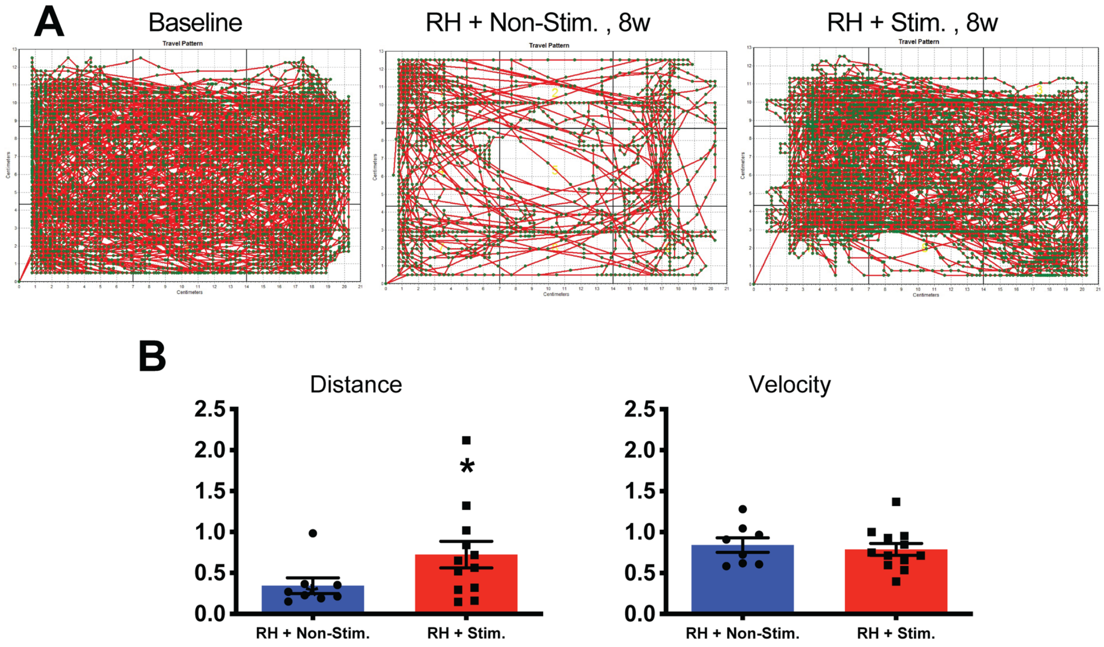

Supplementary Fig 9. Transcranial optogenetic stimulation increases overground locomotion. (**A**) Representative moving traces showed a significant increase in moving distance in the optogenetic stimulation group as compared to the non-stimulation group at 8 weeks after the C5-RH. (**B**) Quantification of moving distances showed a significantly increase in moving distances in the optogenetic stimulation group as compared to the non-stimulation group (normalized as percent of baseline distances). (**C**) No difference was found in moving velocity between the two groups. Data were presented as means ± s.e.m; *n* = 8-12 per group; two-tailed paired Student's t-test; *: $p < 0.05$.

References

1. Walker CL, Fry CME, Wang J, Du X, Zuzzio K, Liu NK, et al. Functional and Histological Gender Comparison of Age-Matched Rats after Moderate Thoracic Contusive Spinal Cord Injury. *Journal of neurotrauma.* 2019.
2. Walker MJ, Walker CL, Zhang YP, Shields LB, Shields CB, and Xu XM. A novel vertebral stabilization method for producing contusive spinal cord injury. *Journal of visualized experiments : JoVE.* 2015(95):e50149.
3. Zhang YP, Walker MJ, Shields LB, Wang X, Walker CL, Xu XM, et al. Controlled cervical laceration injury in mice. *Journal of visualized experiments : JoVE.* 2013(75):e50030.

4. Al-Ali H, Ding Y, Slepak T, Wu W, Sun Y, Martinez Y, et al. The mTOR substrate S6 Kinase 1 (S6K1) is a negative regulator of axon regeneration and a potential drug target for Central Nervous System injury. *J Neurosci.* 2017.
5. Wang X, Liu Y, Li X, Zhang Z, Yang H, Zhang Y, et al. Deconstruction of Corticospinal Circuits for Goal-Directed Motor Skills. *Cell.* 2017;171(2):440-55 e14.
6. Farr TD, and Whishaw IQ. Quantitative and qualitative impairments in skilled reaching in the mouse (Mus musculus) after a focal motor cortex stroke. *Stroke.* 2002;33(7):1869-75.
7. Qu W, Liu NK, Xie XM, Li R, and Xu XM. Automated monitoring of early neurobehavioral changes in mice following traumatic brain injury. *Neural Regen Res.* 2016;11(2):248-56.
8. Wang Y, Wu W, Wu X, Sun Y, Zhang YP, Deng LX, et al. Remodeling of lumbar motor circuitry remote to a thoracic spinal cord injury promotes locomotor recovery. *eLife.* 2018;7.
9. Ayling OG, Harrison TC, Boyd JD, Goroshkov A, and Murphy TH. Automated light-based mapping of motor cortex by photoactivation of channelrhodopsin-2 transgenic mice. *Nature methods.* 2009;6(3):219-24.
10. Harrison TC, Ayling OG, and Murphy TH. Distinct cortical circuit mechanisms for complex forelimb movement and motor map topography. *Neuron.* 2012;74(2):397-409.
11. Chu YX, Jin XM, Parada I, Pesic A, Stevens B, Barres B, et al. Enhanced synaptic connectivity and epilepsy in C1q knockout mice. *Proceedings of the National Academy of Sciences of the United States of America.* 2010;107(17):7975-80.
12. Liu NK, Deng LX, Zhang YP, Lu QB, Wang XF, Hu JG, et al. Cytosolic phospholipase A2 protein as a novel therapeutic target for spinal cord injury. *Annals of neurology.* 2014;75(5):644-58.
